# Multi-omics factor analysis v2 (MOFA+) reveals specific co-variation patterns in women with PMOS that are strongly influenced by obesity

**DOI:** 10.1093/hropen/hoag080

**Published:** 2026-09-08

**Authors:** Edmond Géraud-Aguilar, M Ángeles Martínez-García, María Insenser, Susana Barceló-Cerdá, Manuel Luque-Ramírez, Francisco García-García, Héctor F Escobar-Morreale

**Affiliations:** Department of Applied Statistics and Operational Research and Quality, Universitat Politècnica de València, Valencia, Spain; Diabetes, Obesity and Human Reproduction Research Group, Department of Endocrinology & Nutrition, Hospital Universitario Ramón y Cajal & Universidad de Alcalá & Instituto Ramón y Cajal de Investigación Sanitaria IRYCIS & Centro de Investigación Biomédica en Red Diabetes y Enfermedades Metabólicas Asociadas CIBERDEM, Madrid, Spain; Diabetes, Obesity and Human Reproduction Research Group, Department of Endocrinology & Nutrition, Hospital Universitario Ramón y Cajal & Universidad de Alcalá & Instituto Ramón y Cajal de Investigación Sanitaria IRYCIS & Centro de Investigación Biomédica en Red Diabetes y Enfermedades Metabólicas Asociadas CIBERDEM, Madrid, Spain; Department of Applied Statistics and Operational Research and Quality, Universitat Politècnica de València, Valencia, Spain; Diabetes, Obesity and Human Reproduction Research Group, Department of Endocrinology & Nutrition, Hospital Universitario Ramón y Cajal & Universidad de Alcalá & Instituto Ramón y Cajal de Investigación Sanitaria IRYCIS & Centro de Investigación Biomédica en Red Diabetes y Enfermedades Metabólicas Asociadas CIBERDEM, Madrid, Spain; Computational Biomedicine Laboratory, Príncipe Felipe Research Center (CIPF), Valencia, Spain; Diabetes, Obesity and Human Reproduction Research Group, Department of Endocrinology & Nutrition, Hospital Universitario Ramón y Cajal & Universidad de Alcalá & Instituto Ramón y Cajal de Investigación Sanitaria IRYCIS & Centro de Investigación Biomédica en Red Diabetes y Enfermedades Metabólicas Asociadas CIBERDEM, Madrid, Spain

**Keywords:** androgen excess, metabolomics, microbiota, obesity, proteomics, sexual dimorphism

## Abstract

**STUDY QUESTION:**

What are the mechanisms underlying the complex interactions between polyendocrine metabolic ovarian syndrome (PMOS) and obesity as studied by a multi-omics integration approach?

**SUMMARY ANSWER:**

Multi-Omics Factor Analysis v2 (MOFA+) suggested that PMOS was characterized by an androgen-driven metabolic reprogramming in which sexual dimorphism was reshaped in an obesity- and omics-specific manner, consisting of strong associations of proinflammatory proteins and adipokines with gut microbiota genera and metabolomics variables that were amplified by obesity.

**WHAT IS KNOWN ALREADY:**

PMOS is a common metabolic–endocrine disorder in which androgen excess, weight excess, and obesity complex interactions define a particularly unfavorable cardiometabolic phenotype.

**STUDY DESIGN, SIZE, DURATION:**

Cross-sectional study with 2 control groups involving 46 Caucasian young adults, conducted at an academic hospital in Madrid, Spain.

**PARTICIPANTS/MATERIALS, SETTING, METHODS:**

We recruited 15 women with PMOS, 16 healthy control women, and 15 control men of similar mean age and BMI. Participants were further grouped into non-obese (BMI < 30 kg/m²) or obese (BMI ≥ 30 kg/m²) subjects. We integrated serum metabolomics and circulating proteins with gut microbiota composition, using MOFA+.

**MAIN RESULTS AND THE ROLE OF CHANCE:**

MOFA+ identified five latent factors explaining the main sources of variation across omics layers. Results showed sexual dimorphism in an omics- and obesity-specific manner: the metabolomics profile presented sexual dimorphism in non-obese individuals, whereas circulating proteins and gut microbiota differed between women with or without PMOS and men only in obese subjects. Correlation analyses revealed a global weakening of associations in men, particularly among metabolites, a reinforcement of protein-related associations with obesity, and a distinct PMOS-specific pattern characterized by strengthened correlations within and between gut microbiota and proteomic features. Women with PMOS exhibited the strongest metabolomics–proteomics correlations, further exacerbated by obesity.

**LIMITATIONS, REASONS FOR CAUTION:**

The relatively small sample size of our study might have missed relevant associations that could have reached statistical significance in larger samples. Also, we only included women with hyperandrogenic phenotypes of PMOS. Hence, our results may not apply to non-hyperandrogenic phenotypes of the syndrome.

**WIDER IMPLICATIONS OF THE FINDINGS:**

PMOS showed a metabolic profile similar to that of obesity even in the absence of excess weight, supporting the concept that adipose tissue dysfunction, proinflammatory proteins, and adipokines, rather than fat mass excess *per se*, were a central driver of the metabolic disturbances of the syndrome. Altogether, these findings highlight the complex interplay between androgen excess, adiposity, and host–microbiota interactions and provide a systems-level framework that may facilitate the identification of novel biomarkers and targeted therapeutic strategies in PMOS. Also, these findings highlight the importance of considering sex, sex hormones, and obesity-specific contexts in biomarker discovery and mechanistic studies.

**FUNDING:**

This research was funded by Instituto de Salud Carlos III grants PI11/0357, PI18/01122, PI21/00116 and PI25/00434 and co-funded by the European Union. F.G.-G. was supported by CIAICO/2023/149 and funded by the Consellería de Educación, Cultura y Universidades de la Generalitat Valenciana, and PID2021-124430OA-I00 funded by MICIU/AEI/10.13039/501100011033 and by ERDF/EU. CIBERDEM and IRYCIS are also initiatives of Instituto de Salud Carlos III.

**DISCLOSURES:**

The funding organizations played no role in the study design; collection, analysis, and interpretation of data; the writing of the report; or the decision to submit the report for publication. The authors have no competing interests to disclose.

**TRIAL REGISTRATION NUMBER:**

N/A

WHAT DOES THIS MEAN FOR PATIENTS? Polyendocrine metabolic ovarian syndrome (PMOS)—formerly known as polycystic ovary syndrome or PCOS—increases the risk of cardiovascular and metabolic problems, especially in women with obesity. For a long time, doctors were not sure if this danger came from the syndrome itself or simply from being overweight.

This new study, which performed a combined analysis of previous data obtained regarding metabolites and proteins circulating in blood and the balance of gut bacteria present in women with PMOS, revealed that the main issue is not only the amount of body fat, but how that fat behaves. Instead of just being in excess, the fat tissue becomes inflamed and releases harmful proteins. This poorly functioning fat is one of the big players in the cardiovascular and metabolic risks in women with this condition.

## Introduction

Polyendocrine metabolic ovarian syndrome (PMOS)—formerly known as polycystic ovary syndrome (PCOS)—is a common metabolic–endocrine–reproductive disorder characterized by a combination of androgen excess and/or ovarian dysfunction, which may result into oligo-anovulation and/or polycystic ovaries (Escobar-Morreale, 2018). PMOS is frequently associated with metabolic disturbances, such as increased visceral fat, insulin resistance, and increased risk of developing Type 2 diabetes (Escobar-Morreale, 2018). Obesity further worsens infertility and increases the risk of cardiovascular diseases, amplifying the overall negative impact of the syndrome throughout the life of affected women (Escobar-Morreale, 2018; Millan-de-Meer *et al.*, 2023).

In recent decades, researchers have made significant efforts to understand the complexity of PMOS pathophysiology, recognizing it as a syndrome with multiple causes and a variety of clinical manifestations. However, our understanding of this heterogeneous syndrome remains incomplete, and several critical knowledge gaps have been recently identified (Stener-Victorin *et al.*, 2024). Among the areas requiring further exploration are the molecular mechanisms of insulin resistance, oxidative stress, and mitochondrial dysfunction. Additionally, the key triggers and molecular pathways driving PMOS-specific reproductive and cardiometabolic dysfunction need to be further elucidated. Recent research suggests that women with PMOS have changes in the composition of their gut microbiota that may be linked to a chronic low-grade inflammatory state and may contribute to the metabolic and inflammatory profiles observed in these patients (Insenser *et al.*, 2018; Martinez-Garcia *et al.*, 2024). There is also a need to identify biomarkers for early diagnosis, development of novel drug targets, and predict long-term outcomes.

In previous studies, we fully characterized a cohort of Caucasian young adults, which included women with PMOS, non-hyperandrogenic women with regular menses, and healthy men, subgrouped by the presence or absence of obesity. Our studies involved analyzing hormonal, metabolomic, and proteomic profiles, as well as the composition of gut bacteria (Insenser *et al.*, 2018; Fuertes-Martin *et al.*, 2019; Martinez-Garcia *et al.*, 2019, 2020; Escobar-Morreale *et al.*, 2023). Overall, we found that women with PMOS frequently exhibited fasting and postprandial profiles that resembled those of men and not those of control women, suggesting a certain masculinization of metabolism because of androgen excess. Moreover, obese individuals consistently showed higher circulating markers of inflammation and worse metabolic disturbances compared to non-obese individuals. Similarly, the diversity and composition of the gut microbiota of young adults were influenced by the combined effects of sex, sex hormone concentrations, and obesity, presenting with specific abnormalities in women with PMOS. These studies collectively highlighted the intricate relationships between obesity, sex hormones, and hormonal, metabolic, and inflammatory responses in PMOS.

However, each of our earlier omics studies focused on a single biological level, whereas PMOS, as a multifaceted metabolic–endocrine–reproductive disorder, involves complex biological processes that rarely result from changes at just one molecular level. Integrating multiple data sets from different omics layers may help identify pathways, biomarkers, and other relationships present in pathological processes (Hasin *et al.*, 2017).

Here, we aimed to explore how multi-omics approaches, combined with clinical data, could help uncover the mechanisms underlying PMOS. We focused on how different omics profiles showed the effects of sex, obesity, and PMOS in young adults and whether these influences overlapped or aligned. Additionally, we aimed to assess the effectiveness of multi-omics integration in identifying specific biomarkers for PMOS.

## Materials and methods

### Study cohort and data collection

This is the final report of a broader study that addressed the role of sex, PMOS, and obesity on the fasting and postprandial metabolism in young adults. For the present study, we used fasting samples analyzed from 46 Caucasian young adult volunteers, including 15 patients with PMOS, 16 control women, and 15 control men. Subjects were selected to ensure that the three groups were similar in terms of age and BMI and were further classified into non-obese (BMI < 30 kg/m², n = 23) and obese (BMI ≥ 30 kg/m², n = 23) subgroups. The diagnosis of PMOS required the presence of clinical (modified Ferriman–Gallwey score > 8) and/or biochemical hyperandrogenism (total testosterone > 2.3 nmol/l, calculated free testosterone >35 pmol/l, androstenedione > 13.8 nmol/l and/or dehydroepiandrosterone sulfate > 9.2 µmol/l), menstrual dysfunction, and the exclusion of other disorders (Zawadzki and Dunaif, 1992). We did not include non-hyperandrogenic phenotypes of PMOS because the study aimed to address the metabolic effects of androgen excess in women. Control women showed no evidence of hyperandrogenism or ovulatory dysfunction.

Thus, all patients exhibited the classic PMOS phenotype (Zawadzki and Dunaif, 1992), while no control women met the criteria for PMOS according to any current classification. Before enrollment, participants had no history of obesity-associated comorbidities, including glucose tolerance disorders, hypertension, sleep apnea, cardiovascular disease, or male hypogonadism. None of the subjects presented smoking habits or had received treatment with oral contraceptives, antiandrogens, sex steroids, insulin sensitizers, or drugs that might interfere with clinical or biochemical variables for at least 6 months before sampling. The study was conducted in accordance with the Declaration of Helsinki and approved by the Ethics Committee of the Hospital Universitario Ramón y Cajal on 4 November 2011 (PI11/00357). All participants provided their written informed consent to participate in the study.

Technical characteristics of the methodology and assays used for the phenotyping of participants, as well as the statistical analyses used for their comparisons among subgroups, the profiling of circulating proteins and metabolomics in serum, as well as the gut microbiota analyses from fecal samples, have been described in detail in our previous studies (Insenser *et al.*, 2018; Fuertes-Martin *et al.*, 2019; Martinez-Garcia *et al.*, 2019, 2020; Escobar-Morreale *et al.*, 2023). As this study was part of a broader project addressing postprandial metabolism changes as a whole, sample size calculation was based on previous data from Gonzalez *et al.* (2006) reporting differences between patients with PMOS and control women in the percentage change of nuclear factor kappa B expression in mononuclear cells after a standard oral glucose tolerance test. We used the GRANMO version 7.12 online sample size and power calculator (DataRUS, LARS Services, Barcelona, Spain; https://www.datarus.eu/aplicacion/granmo/). Setting alpha at 0.05 and beta at 0.2 for a two-sided test, the inclusion of eight individuals per group would allow detection of a mean difference in percentage change of 50.4%, assuming a standard deviation of 34.1%.

### Multi-omics factor data integration and downstream statistical analysis

We applied Multi-Omics Factor Analysis v2 (MOFA+) within a hybrid framework combining unsupervised exploratory modeling (Argelaguet *et al.*, 2020) with downstream phenotype-driven confirmatory analysis. The dataset included 278 unfiltered features: 38 serum metabolites (Escobar-Morreale *et al.*, 2023), 27 circulating proteins (Fuertes-Martin *et al.*, 2019; Martinez-Garcia *et al.*, 2019, 2020, 2021, 2024), and 213 gut microbiota taxonomic groups (Insenser *et al.*, 2018). Metabolomics and proteomics data were scaled and centered. Gut microbiota data were normalized using a Dirichlet multinomial model (ALDEx2), satisfying Gaussian likelihood assumptions (Fernandes *et al.*, 2014). No missing values or outliers were detected.

MOFA was optimized using the Evidence Lower Bound (ELBO) across 4–12 latent factors (10 seeds), with orthogonality verified via diagnostic correlation plots (*X ≈ ZW^T^*). Features were filtered using a two-stage selection strategy:

1. Latent Factor Loadings: Features exceeding a factor loading threshold (|*W*| > 0.6) were selected, and signal variation was isolated by reconstructing data (*X^(reconstructed)^ = Z_f_W_f_^T^*).
2. Bayesian Differential Analysis: Reconstructed features were evaluated against clinical groups (obesity and subgroups) using a Bayesian two-sample *t*-test framework with Benjamini–Hochberg correction (adjusted *P* < α).

This strategy retained all metabolomic and proteomic features and eight microbiota taxa. Post-selection downstream analyses included empirical Bayesian *t*-tests, two-way analysis of variance, Pearson’s correlations, and Principal Component Analysis (PCA). The detailed description of these analyses may be found in the Supplementary Materials and Methods. All analyses were performed in R v4.4.2 (R Foundation for Statistical Computing, Vienna, Austria; https://www.R-project.org/) with statistical significance set at *P *< 0.05. Code was hosted at https://github.com/eddgeag/integromics_2.

## Results

### Baseline characteristics of study subjects

The clinical, hormonal, and metabolic characteristics of participants at fasting are shown in Table 1. According to sex, men showed higher total and free testosterone levels and waist-to-hip ratio (WHR) than both groups of women, but lower levels of fat mass percentage, estradiol and sex hormone-binding globulin (SHBG). Women with PMOS had higher hirsutism scores and circulating androgens than non-hyperandrogenic control women, but no statistically significant differences in terms of waist circumference (WC) or WHR. In addition, we did not observe significant differences in metabolic parameters between men and women or among control women and participants with PMOS, except for lower mean HDL-cholesterol values in men. Obese individuals, regardless of sex and PMOS, showed increased BMI, WC, WHR, fat mass percentage, total and free estradiol values, free testosterone, fasting glucose, insulin, and homeostasis model assessment of insulin resistance (HOMA-IR), and decreased insulin sensitivity index (ISI) and SHBG concentrations. Also, no differences among groups were observed in acquired factors that could influence gut microbiota, such as recent antibiotic use, periodontal or dental disease, or urinary or gynecological infections.

**Table 1.** Clinical, hormonal, and metabolic characteristics of participants.

	Control women		Women with PMOS		Men		Group *P value*	Obesity *P value*	Interaction *P value*
	Non-obese	Obese	Non-obese	Obese	Non-obese	Obese			
	n = 8	n = 8	n = 7	n = 8	n = 8	n = 7			
Age (yr)	27.3 ± 4.0	27.3 ± 6.5	23.0 ± 7.9	29.9 ± 5.1	23.3 ± 3.3	23.6 ± 3.2	0.116	0.129	0.138
BMI (kg/m^2^)	23.4 ± 1.9	35.9 ± 4.2	24.4 ± 2.8	37.0 ± 4.8	22.9 ± 2.0	34.6 ± 3.4	0.309	<0.001	0.888
Waist to hip ratio*^,^^†^	0.74 ± 0.10	0.83 ± 0.12	0.74 ± 0.04	0.85 ± 0.10	0.84 ± 0.04	0.90 ± 0.10	0.005	<0.001	0.542
Waist circumference (cm)	75.2 ± 8.4	100.4 ± 17.4	72.9 ± 8.00	104.6 ± 10.5	81.0 ± 5.6	110.9 ± 14.4	0.113	<0.001	0.717
Fat mass percentage (%)*^,^^†^	34.8 ± 5.6	43.7 ± 5.7	31.5 ± 6.5	42.9 ± 3.9	15.5 ± 5.2	33.6 ± 7.4	<0.001	<0.001	0.095
Hirsutism score	1.3 ± 1.3	1.8 ± 1.2	10.1 ± 5.1	9.3 ± 4.5	NA		<0.001	0.887	0.589
Total testosterone (nmol/l)*^,^^†^	1.6 ± 0.3	2.0 ± 0.5	2.5 ± 0.7	2.4 ± 1.0	19.2 ± 3.4	16.6 ± 2.8	<0.001	0.993	0.134
FT (pmol/l)*^,^^†^	20 ± 5	31 ± 8	38 ± 10	45 ± 24	462 ± 106	464 ± 81	<0.001	0.012	0.022
Total estradiol (pmol/l)*^,^^†^	153 ± 66	276 ± 200	188 ± 230	149 ± 49	64 ± 13	95.8 ± 25.3	<0.001	0.024	0.598
FE_2_ (pmol/l)*	2.7 ± 1.2	5.3 ± 3	3.9 ± 4.9	3.5 ± 1.4	1.7 ± 0.5	2.7 ± 0.7	0.027	0.003	0.549
Ratio FT/FE^‡^	8.3 ± 2.6	7.5 ± 4.6	19.0 ± 12.7	14 ± 5.4	286.2 ± 60.4	179.0 ± 39.3	<0.001	0.087	0.272
SHBG (nmol/l)^‡^	60 ± 24	43 ± 14	44 ± 18	32 ± 13	28 ± 10	18 ± 5	<0.001	0.007	0.788
DHEAS (μmol/l)*	5.1 ± 0.9	5.3 ± 1.5	8.5 ± 3.9	5.7 ± 2.1	6.5 ± 1.8	8.5 ± 3.2	0.025	0.798	0.027
Androstenedione (nmol/l)^‡^	9.1 ± 3.1	9.4 ± 2.8	16.1 ± 3.5	13.3 ± 5.9	7.3 ± 1.8	9.8 ± 4.2	<0.001	0.992	0.175
Total cholesterol (mmol/l)	4.5 ± 0.9	4.7 ± 1.2	4.4 ± 1.2	4.4 ± 1.0	3.9 ± 0.6	4.6 ± 0.8	0.635	0.263	0.607
LDL-cholesterol (mmol/l)	2.8 ± 0.7	2.8 ± 0.4	2.6 ± 1.0	2.6 ± 1.0	2.4 ± 0.5	3.1 ± 0.6	0.811	0.263	0.291
HDL-cholesterol (mmol/l)*^,^^†^	1.3 ± 0.3	1.2 ± 0.2	1.4 ± 0.2	1.2 ± 0.2	1.2 ± 0.2	1.0 ± 0.1	0.011	0.020	0.619
Triglycerides (mmol/l)	0.8 ± 0.2	0.9 ± 0.4	0.8 ± 0.2	1.2 ± 0.6	0.9 ± 0.3	1.2 ± 0.6	0.423	0.055	0.755
Fasting glucose (mmol/l)^‡^	4.7 ± 0.3	5.6 ± 0.4	4.8 ± 0.4	4.9 ± 0.4	5.3 ± 0.5	5.3 ± 0.5	0.014	0.014	0.039
Fasting insulin (pmol/l)	53 ± 28	92 ± 45	47 ± 35	83 ± 28	37 ± 14	76 ± 28	0.383	<0.001	0.969
HOMA-IR	1.6 ± 0.9	3.3 ± 1.6	1.5 ± 1.1	2.6 ± 0.9	1.3 ± 0.5	2.6 ± 1.0	0.348	<0.001	0.780
Insulin sensitivity index	7.0 ± 2.7	3.3 ± 1.2	8.2 ± 5.2	3.5 ± 1.4	7.6 ± 2.9	3.6 ± 1.7	0.899	<0.001	0.997
Antibiotics	0 (0)	0 (0)	0 (0)	2 (25)	1 (12.5)	0 (0)	0.217	1.000	NA
Periodontal or dental disease	0 (0)	1 (12.5)	0 (0)	0 (0)	0 (0)	0 (0)	0.434	1.000	NA
Urinary infection	0 (0)	0 (0)	0 (0)	1 (12.5)	0 (0)	0 (0)	0.434	1.000	NA
Vaginal candidiasis or infection	0 (0)	0 (0)	0 (0)	1 (12.5)	NA		0.396	1.000	NA

DHEAS, dehydroepiandrosterone-sulfate; FE_2_, free estradiol; FT, free testosterone; HOMA-IR, homeostasis model assessment of insulin resistance; PMOS, polyendocrine metabolic ovarian syndrome; NA, not applicable; SHBG, sex hormone-binding globulin. Data are means ± SD or counts (percentage). The effects of group and obesity on continuous variables were analyzed by a two-way GLM after applying a two-step transformation as needed to ensure a normal distribution of the variables.

* *P *< 0.05 for the differences between men and control women.

† *P *< 0.05 for the differences between men and women with PMOS.

‡ *P *< 0.05 for the differences between women with PMOS and control women, regardless of obesity. For categorical variables, differences between groups were analyzed by χ^2^ tests. Reproduced with permission from Insenser *et al.* (2018), Copyright © 2018 Endocrine Society.

### Multi-omics data analysis revealed potential patterns of co-variation

**Figure 1.**
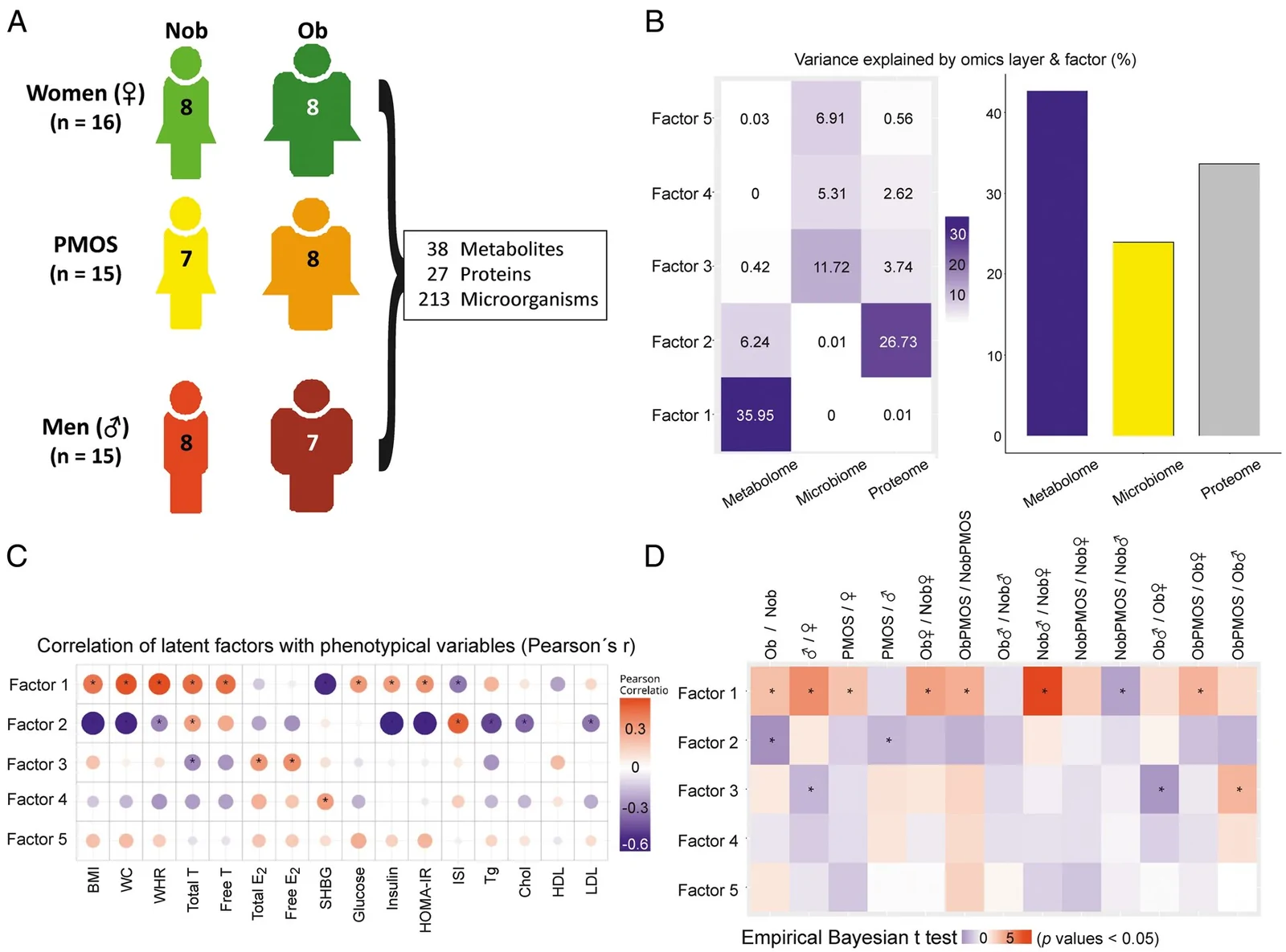
**Overview of multi-omics integration by Multi-Omics Factor Analysis v2 (MOFA+) and latent factors differences.** (**A**) Groups of subjects and features analyzed in each omics layer. (**B**) Proportion of variance explained by the latent factors of the MOFA+ model. The factors represent latent components that capture variability, either shared across different omics layers or specific to each one. (**C**) Heatmap of Pearson’s correlation coefficients between the latent factors and clinical variables. (**D**) Heatmap of empirical Bayesian *t*-tests comparing the differences in latent factors between study groups. Subjects are categorized by obesity (Nob, non-obese; Ob, obese) and group [control women (♀), women with polyendocrine metabolic ovarian syndrome (PMOS), and men (♂)]. Each cell represents the *t*-statistic for the corresponding comparison. Red and blue cells indicate higher and lower values in the first group of the comparison, respectively. *P*-values are corrected using the Benjamini–Hochberg procedure. *P < 0.05.

To analyze in depth the complex interplay between metabolites, circulating proteins, and gut microbiota in PMOS, while accounting for the effects of sexual steroids and obesity, we conducted an integrative multi-omics analysis of a well-characterized cohort (Fig. 1A).

The MOFA+ model initially revealed five latent factors that capture the primary sources of shared biological or technical variation across multiple omics data modalities, with the plasma metabolome explaining most of the variance (43%), followed by the circulating proteomic profile (33%) and the gut microbiota composition (24%) (Fig. 1B). Latent factors derived from MOFA+ captured integrative patterns across these omics layers. The contribution of each feature to the factors is depicted in Supplementary Fig. S1, which shows the weights of the variables contributing to each factor.

Based on statistical significance and the proportion of total variance explained within each omics layer, Factors 1 and 2 accounted for nearly 70% of the total variance. Factor 1 was driven almost exclusively by the metabolome (with the particular contribution of 1-methylhistidine, glucose, lysine, valine, and 2-oxoisocaproic and 2-oxoisovaleric acids), whereas Factor 2 captured the greatest co-variation of the proteomic profile (all glycoproteins heavily weighted with negative values), with a lesser contribution from the metabolomic data (Fig. 1B; Supplementary Fig. S1). In contrast, the contribution of gut microbiota was observed only in Factors 3, 4, and 5, with a more modest contribution of the proteomic profile (Fig. 1B).

To further characterize the biological relevance of these latent factors, we investigated their associations with clinical and biochemical phenotypical variables. Panel C of Fig. 1 illustrates the relationship between each factor and measures related to adiposity, lipid and glucose metabolism, and sex hormones. Factor 1 showed statistically significant positive correlations with BMI, WC, WHR, total and free testosterone, glucose, insulin, and HOMA-IR, while showing a negative association with SHBG and ISI. In contrast, an almost opposite pattern was found for Factor 2 in its association with several variables: although its correlations with total testosterone and ISI were positive, it showed negative correlations with BMI, WC, WHR, insulin, HOMA-IR, triglycerides, and total and LDL cholesterol. Factor 3 showed positive correlations with total and free estradiol concentrations, along with a negative correlation with total testosterone. Lastly, Factor 4 was positively correlated with SHBG.

To explore how the identified latent factors related to the different groups of subjects defined by their sex hormone profiles (control women, women with PMOS, and control men) and by the presence of obesity (obese vs non-obese subjects), we performed empirical Bayesian *t*-tests (Fig. 1D). Panel D of Fig. 1 presents a heatmap summarizing the statistical relationships between latent factors and the different subgroups of young adults. Factor 1 was different among obese and non-obese subjects when considering all subjects as a whole and in both groups of women. Also, differences were observed when considering control men and control women as a whole, suggesting sexual dimorphism, with this difference being more evident in non-obese subjects (Fig. 1D). Factor 2 was different between obese and non-obese subjects, regardless of sex and sex hormones and was also different between women with PMOS and men, regardless of obesity. Factor 3 showed differences between men and control women, particularly in those with obesity, which may suggest sexual dimorphism in gut microbiota composition considering the major contribution of gut bacteria to this factor (Fig. 1B; Supplementary Fig. S1). This factor was also different between obese women with PMOS and obese men. Factors 4 and 5 did not show significant differences between any group of subjects (Fig. 1D).

### Multi-omics group differences in circulating metabolites, proteins, and gut bacteria

**Figure 2.**
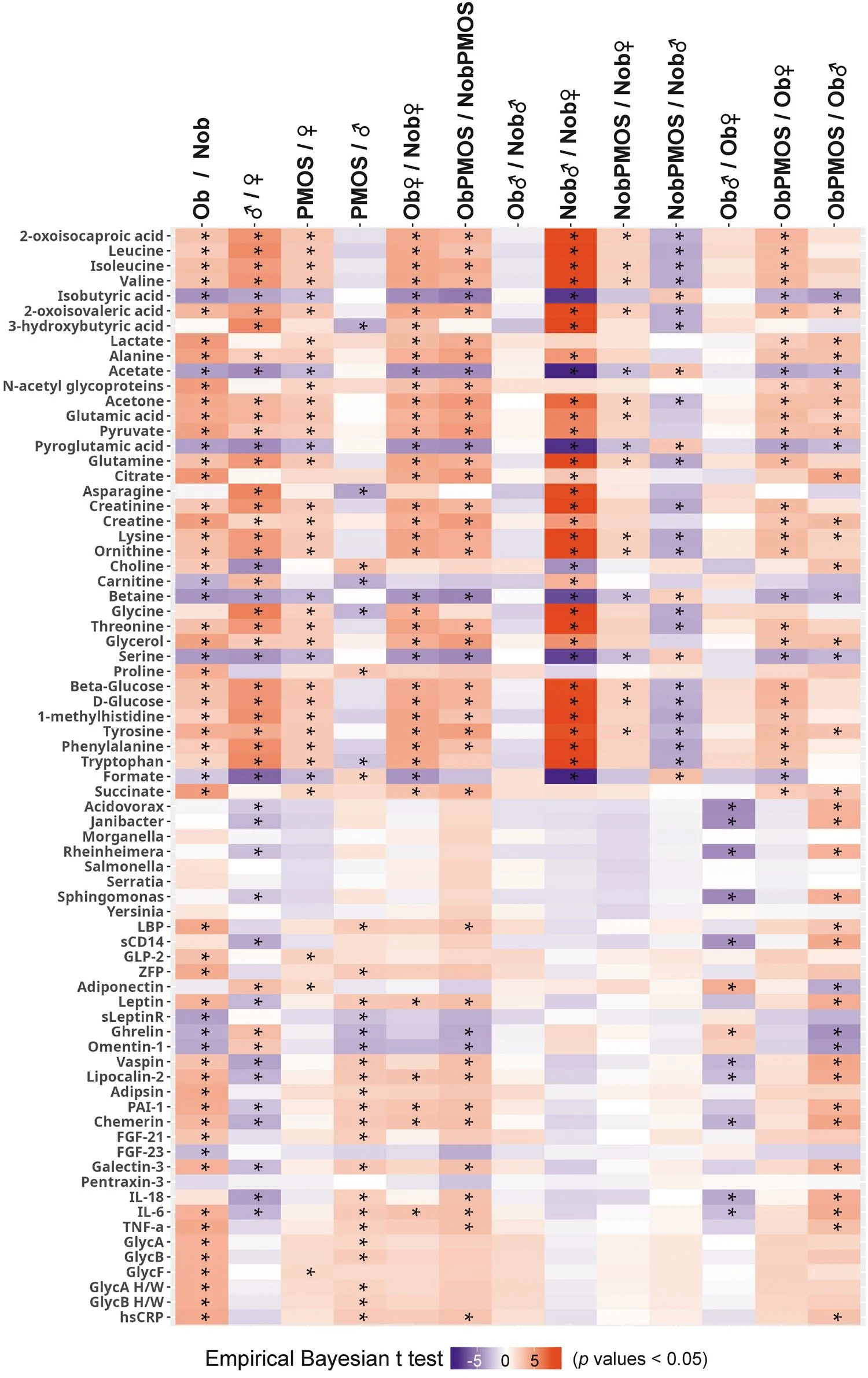
**Heatmap of empirical Bayesian *t*-tests of the features derived from the reconstructed signal based on the Multi-Omics Factor Analysis v2 (MOFA+) integration model**. The heatmap shows differences in variables between groups of subjects categorized by obesity Subjects are categorized by obesity (Nob, non-obese; Ob, obese) and group [control women (♀), women with polyendocrine metabolic ovarian syndrome (PMOS), and men (♂)]. Each cell represents the *t*-statistic for the corresponding comparison. Red and blue cells indicate higher and lower values in the first group of the comparison, respectively. *P*-values are corrected using the Benjamini–Hochberg procedure. *P < 0.05.

We performed empirical Bayesian *t*-tests to assess the individual contribution of each feature in subgroup comparisons. Subjects were categorized by obesity and by sex- and sex hormones subgroups (control men, control women, and PMOS). Each cell represents the *t*-statistic for the comparison, with an asterisk denoting statistically significant differences (Fig. 2).

#### Influence of obesity

When we compared obese and non-obese subjects regardless of the subgroups defined by sex- and sex hormones, obesity was predominantly associated with higher levels of circulating metabolites and proteins, while gut bacterial composition remained unchanged (Fig. 2). However, a subset of variables showed decreased levels in obesity, including isobutyric acid, acetate, pyroglutamic acid, carnitine, serine, betaine, formate, as well as soluble leptin receptor (sLepR), ghrelin, omentin, and fibroblast growth factor (FGF)-23 (Fig. 2). Such differences were actually dependent on women’s subgroups, as no differences were found between obese and non-obese men (Fig. 2). In obese women with PMOS compared with their non-obese counterparts, we observed a specific pattern consisting of an increase in lipopolysaccharide binding protein (LBP), vaspin, high-sensitivity C-reactive protein (hsCRP), interleukin (IL)-18, galectin-3, and tumor necrosis factor alpha (TNFα), and a decrease in omentin and ghrelin (Fig. 2).

#### Influence of sex

A consistent pattern of sexual dimorphism was observed across all three omics layers, with clear differences between male and female controls (Fig. 2). When stratifying by obesity, these sex-related differences became omics-specific. In non-obese subjects, the heatmap showed the largest sex-related differences in almost all circulating metabolites, but differences in gut microbiota and proteins disappeared. In contrast, obese subjects showed sex-related differences exclusively in gut bacteria composition and circulating proteins (Fig. 2). In particular, *Acidovorax*, *Janibacter*, *Rheinheimera*, and *Sphingomonas* were more abundant in obese control women and obese women with PMOS compared with male controls and also showed higher circulating levels of soluble cluster of differentiation 14 (sCD14), vaspin, lipocalin-2, chemerin, IL-18, and IL-6 and reduced adiponectin and ghrelin (Fig. 2).

#### Influence of PMOS

In the comparison between women with and without PMOS, we found differences in 32 out of 38 metabolites. Most of them increased in patients with PMOS compared with control women, while only isobutyric and pyroglutamic acid, serine, betaine, acetate, and formate were decreased (Fig. 2). These differences were particularly frequent and stronger within the obese subgroup (Fig. 2). Regarding the proteomic layer, we observed modest differences between women with PMOS and non-hyperandrogenic female controls, with only glucagon-like peptide-2 (GLP-2) and GlycF being increased in PMOS patients regardless of obesity (Fig. 2). Nevertheless, gut microbiota remained largely unaffected by PMOS (Fig. 2).

We also observed a partially androgenized pattern in women with PMOS, especially at the metabolomic level because the metabolic profile of women with PMOS resembled that of men (Fig. 2). Only 8 of the 38 metabolites were different between women with PMOS and men, including 3-hydroxybutyric acid, asparagine, and tryptophan (Fig. 2). In contrast, circulating proteins showed a distinctive profile in women with PMOS compared with men, characterized by increased levels of most inflammatory proteins and leptin, and decreased levels of ghrelin, sLepR, and omentin (Fig. 2). Such differences actually occurred at the expense of obese subjects, because non-obese participants maintained most of the sex-related differences in metabolites and did not show differences between women with PMOS and men in any proteomic variable (Fig. 2).

### Correlation analyses

**Figure 3.**
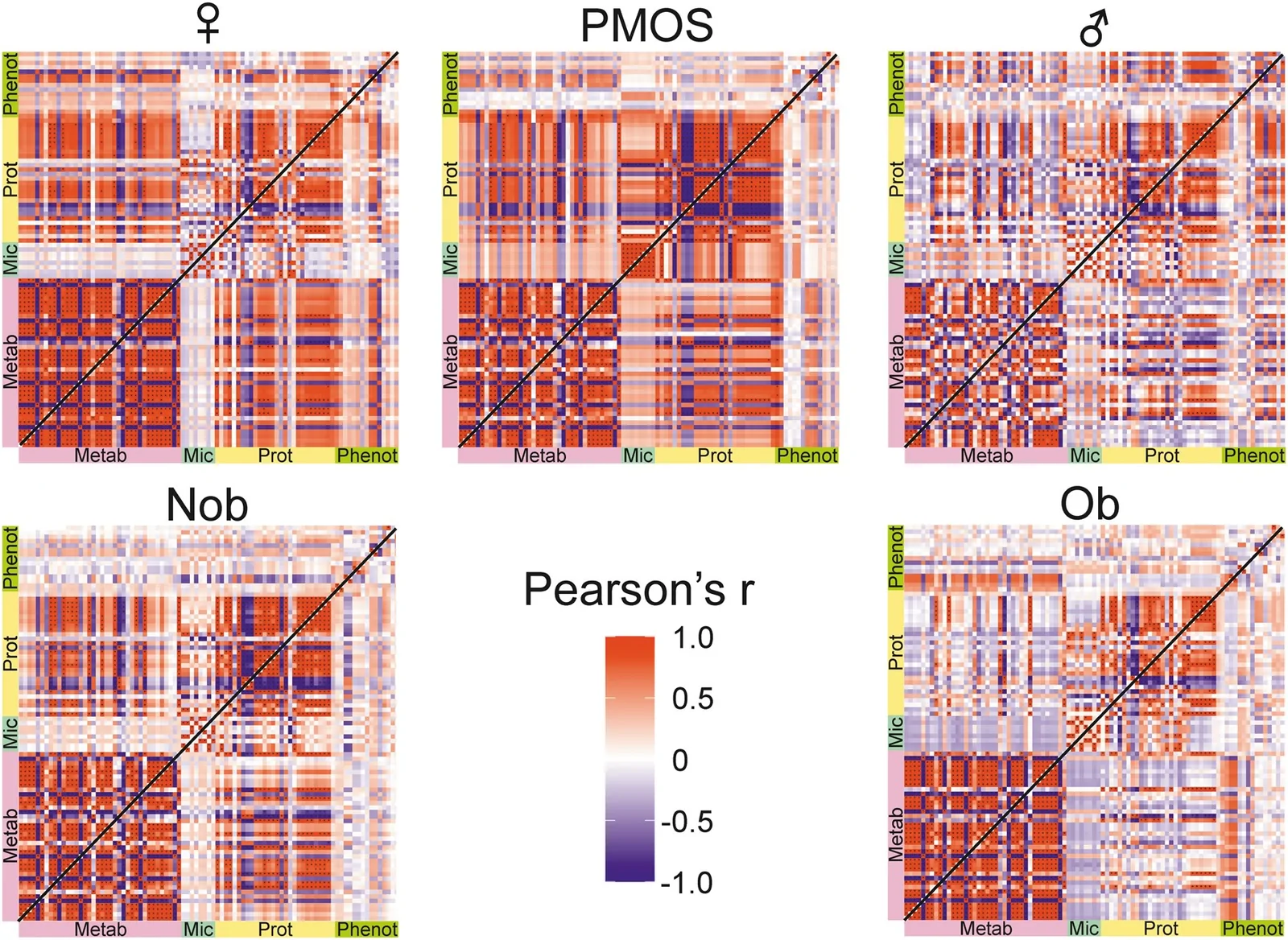
**Correlation matrixes of the features derived from the reconstructed signal based on the Multi-Omics Factor Analysis v2 (MOFA+) integration model**. Features are grouped by omics layer and subjects are categorized by obesity (Nob, non-obese; Ob: obese) and group [control women (♀), women with polyendocrine metabolic ovarian syndrome (PMOS), and men (♂)]. Each cell represents the Pearson’s correlation coefficient between two features.

To explore relationships across and within omics layers, we performed correlation analysis between all molecules, which are represented as heatmaps. The heatmaps of control women, women with PMOS and men, and of obese and non-obese subjects considered as wholes, are depicted in Fig. 3 (these heatmaps did not include the specific molecules and genera in the *x* and *y* axes because of space constraints; those variables may be read in the larger-resolution heatmaps depicted in Supplementary Figs S2, S3, S4, S5, and S6). Heatmaps showed overall similarities among control women and non-obese subjects and between men and obese individuals, with PMOS showing a specific heatmap pattern with no resemblance to those of other groups (Fig. 3).

Considering female sex and absence of obesity as the default for healthy cardiometabolic health (Escobar-Morreale *et al.*, 2014; Strack *et al.*, 2022), the pattern associated with both male sex and obesity was an overall weakening of correlations among variables, particularly among the metabolomic and proteomic variables (Fig. 3). On the contrary, the specific pattern of PMOS consisted in a reinforcement of the correlations among microbiota and proteomic variables (Fig. 3). Of note, the almost-perfect correlations observed among gut microbiota variables suggested a loss of diversity in gut microbiota composition and the existence of a tight ecological and metabolic environment in the microbiota of these women (Insenser *et al.*, 2018) that was not explained by either sex or obesity (Fig. 3).

**Figure 4.**
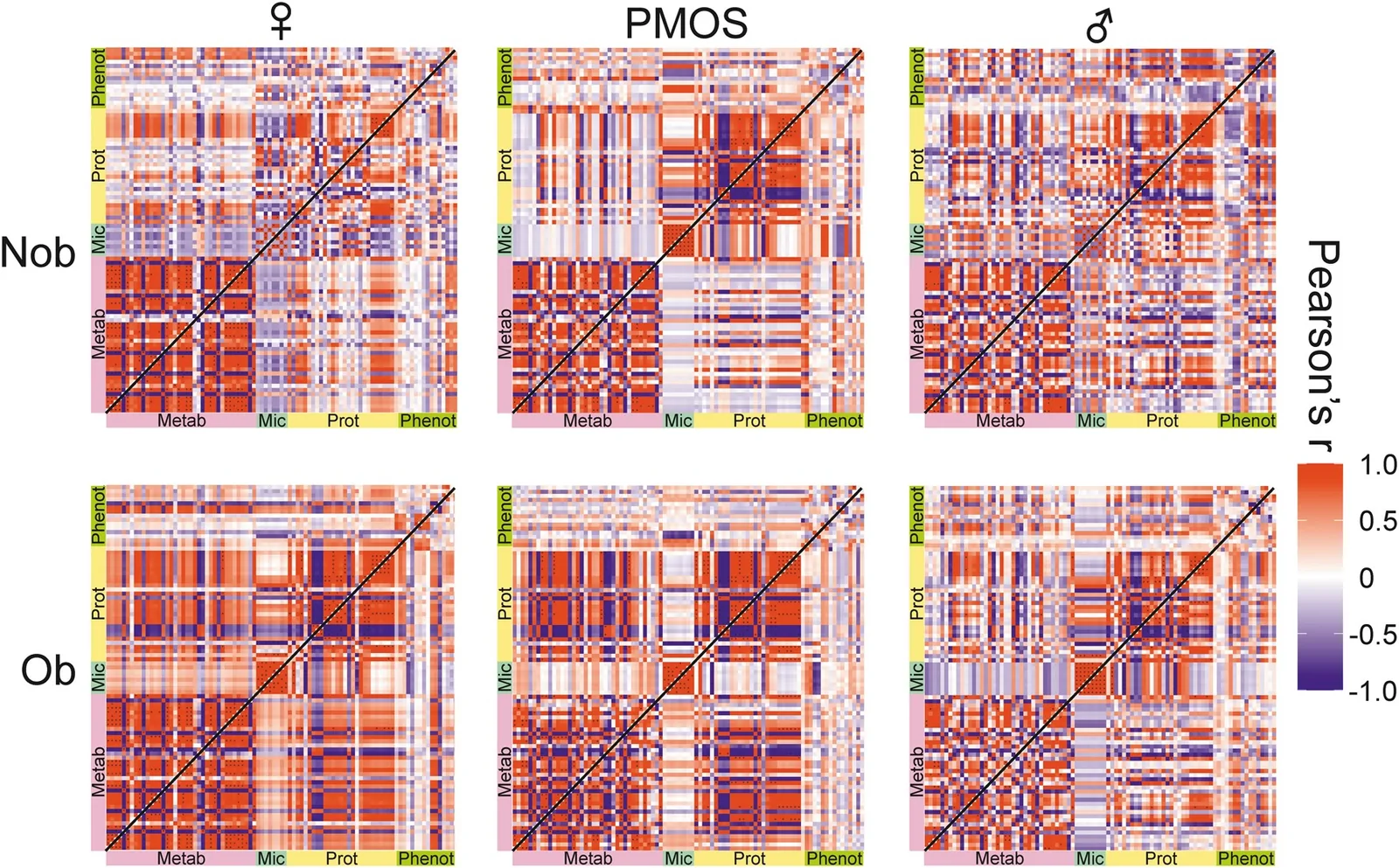
**Correlation matrixes of the features derived from the reconstructed signal based on the Multi-Omics Factor Analysis v2 (MOFA+) integration model in control women (♀), women with polyendocrine metabolic ovarian syndrome (PMOS), and men (♂) stratified by obesity (Nob: non-obese; Ob: obese)**. Features are grouped by omics layer. Each cell represents the Pearson’s correlation coefficient between two features.

The whole picture changed when assessing the correlation heatmaps resulting from the interaction between obesity and the subgroups defined by sex and sex hormones (Fig. 4, variable names depicted in Supplementary Figs S7, S8, S9, S10, S11, and S12). When restricting the analysis to non-obese subjects, sexual dimorphism still consisted of men showing weaker associations among metabolites compared with control women, yet the association among proteomic variables and those of proteomic and metabolomic variables was somewhat stronger in men (Fig. 4). Compared with control women, women with PMOS shared with men the weaker associations among metabolites, as well as the reinforcement of associations among proteomic variables, which were, in fact, the strongest among the three subgroups defined by sex and sex hormones (Fig. 4). Unlike control men and women, non-obese women with PMOS maintained the almost perfect correlation among gut microbiota variables described above when analyzing patients with PMOS as a whole (Figs 3 and 4).

These sex- and sex-hormone-related changes were also found when analyzing obese individuals, with obesity reinforcing the associations among proteins and with metabolomic variables, particularly in the subgroup of obese control women, to the extent that their heatmap was very close to that of PMOS women studied as a whole (Figs 3 and 4). Obese control women, compared with their non-obese counterparts, presented a marked change in gut bacteria associations, among them and with metabolomic features: the associations with metabolites frequently changed from negative to positive, and these women now shared the almost perfect correlations among gut genera with women with PMOS and obese men (Fig. 4). Notably, the amino acid proline showed consistent associations with inflammatory markers, gut permeability markers, and adipokines across all subgroups (Supplementary Figs S7, S8, S9, S10, S11, and S12). These proline associations were particularly numerous and strong in obese women regardless of PMOS and were also present, yet with less proteins, in non-obese women with PMOS and in obese men (Supplementary Figs S7, S8, S9, S10, S11, and S12).

Gut microbiota genera correlated with several proteins, but not with metabolites (Supplementary Figs S2, S3, S4, S5, S6, S7, S8, S9, S10, S11, and S12). *Acidovorax*, *Janibacter*, *Rheinheimera*, and *Sphingomonas* on the one hand, and *Morganella*, *Salmonella*, *Serratia*, *and Yersinia* on the other, behaved as two distinct blocks and correlated with specific proteins in the opposite direction when considering the subgroups defined by sex, sex hormones, and obesity. These proteins included sCD14, sLepR, adiponectin, FGF-23, pentraxin-3, and IL-18 (Figs 3 and 4;  Supplementary Figs S2, S3, S4, S5, S6, S7, S8, S9, S10, S11, and S12). Of note, women with PMOS, regardless of obesity, presented the strongest positive correlation with sCD14, and the strongest negative correlations with adiponectin and pentraxin-3 (Supplementary Fig. S3).

In summary, correlation heatmaps revealed sexual dimorphism in the associations across omics layers with weaker metabolite-related associations in men, a certain effect of obesity consisting of reinforcement of the associations involving proteins, and a specific effect of PMOS that puts them in line with obese controls regarding the associations among metabolites, proteins, and gut microbiota (Fig. 4).

### MOFA+ and PCA

**Figure 5.**
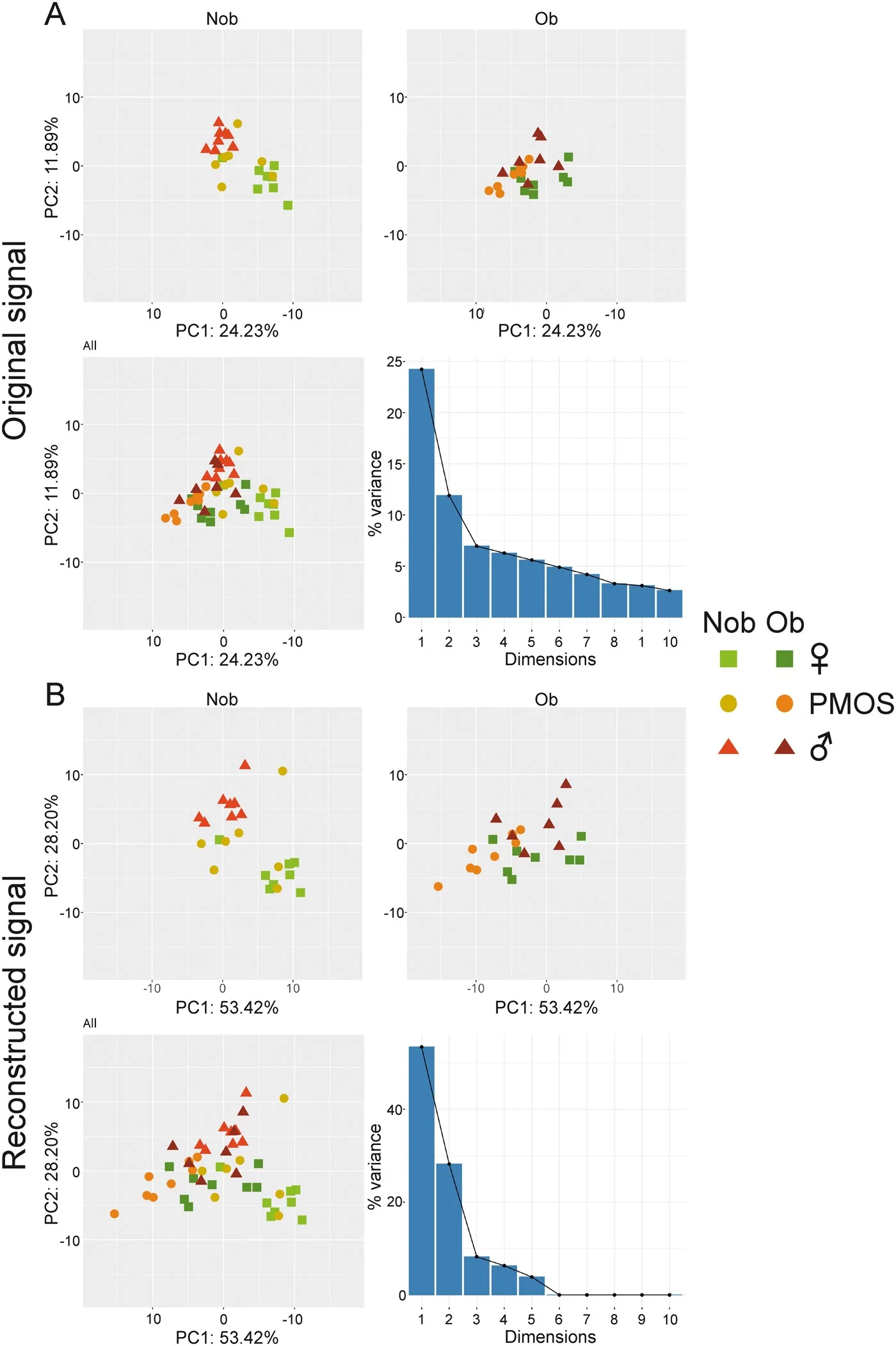
**Principal component analysis (PCA) of the original signal and the Multi-Omics Factor Analysis v2 (MOFA+) reconstructed signal**. The PCA shows the distribution of individual subjects based on the original signal (**A**, upper panels) and the reconstructed signal derived from the MOFA+ integration model (**B**, lower panels). Samples are represented according to subject subgroup: control women (♀); women with polyendocrine metabolic ovarian syndrome (PMOS); men (♂); Non-obese (Nob); and Obese (Ob). Each dot corresponds to an individual sample.

PCA was conducted before (Fig. 5A) and after (Fig. 5B) MOFA+ integration. The percentage of variance explained by the first and second dimensions of PCA increased markedly after MOFA+ integration, accounting for more than 80% of the variance and improving the separation of the subgroups of subjects (Fig. 5). Supplementary Fig. S13 includes the molecules contributing to both PC1 and PC2 along with their weights.

**Figure 6.**
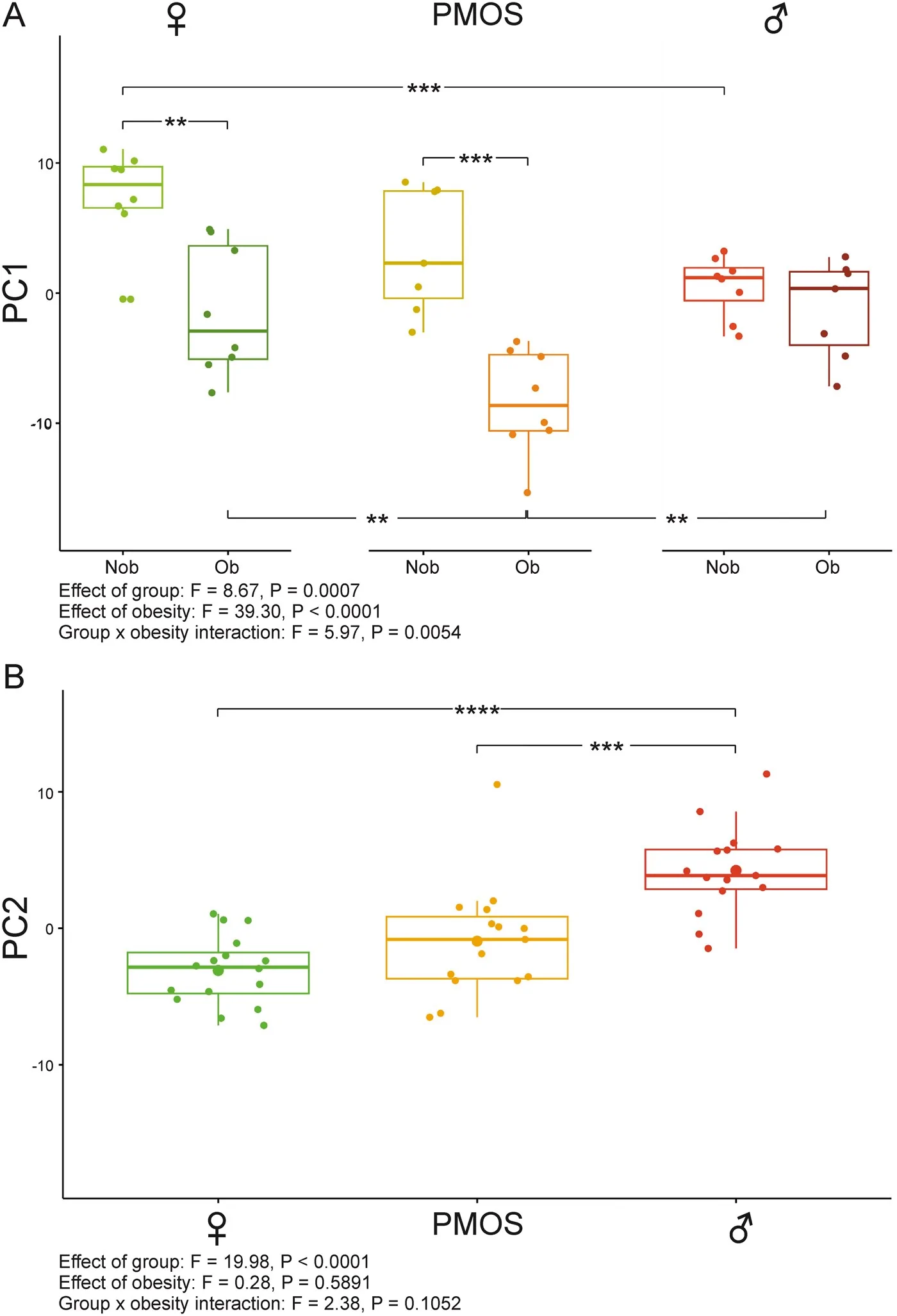
**Differences in principal component analysis (PCA) scores of the Multi-Omics Factor Analysis v2 (MOFA+) reconstructed signal in study groups**. (**A**) PC1 scores of study groups stratified by obesity. (**B**) PC2 scores of subjects subgroups regardless of obesity. Dots represent individual samples. Samples are represented according to subject subgroup: control women (♀); women with polyendocrine metabolic ovarian syndrome (PMOS); men (♂); non-obese (Nob); obese (Ob). Statistical differences were assessed by two-way analysis of variance followed by the Benjamini–Hochberg procedure. *P < 0.05, **P < 0.01, ***P < 0.001, and ****P < 0.0001.

We further characterized the features that contributed to the separation of groups for each dimension of PCA (PC1 and PC2), focusing on the group of subjects and obesity as independent variables and their interaction (Fig. 6).

Such an interaction indicated differences in PC1 between obese and non-obese subjects in control women and in women with PMOS that were not found in men (Fig. 6A). Within non-obese subjects, PC1 was reduced in men compared with control women, with patients with PMOS showing intermediate values that were not different when compared with both control men and women (Fig. 6A). Focusing on obese individuals, PC1 was reduced in women with PMOS compared with both control women and men (Fig. 6A).

The impact of obesity in women with PMOS, unlike that in control women, was related not only to metabolites related to insulin resistance such as β-glucose, leucine, isoleucine, valine, and lysine but also to proteins involved in inflammatory pathways—including IL-6, leptin, vaspin, chemerin, adipsin, GlycA, GlycB, GlycF, and hsCRP (Supplementary Fig. S14)—among others. Such proteins weighted heavily in the comparisons among obese patients with PMOS, obese control women, and obese men (Supplementary Fig. S14). Gut microbiota taxa such as *Acidovorax*, *Janibacter*, and *Sphingomonas* also contributed to the differences between obese women with PMOS and their non-obese counterparts and with obese men (Supplementary Fig. S14). Of note, the comparison of non-obese men and women showed an orderly contribution of the weights of metabolites, proteins, and microbiota genera, suggesting sexual dimorphism in these variables (Supplementary Fig. S14).

For PC2, only the subgroups defined by sex and sex hormones showed differences that occurred regardless of obesity and consisted of increased PC2 in men compared to both groups of women (regardless of PMOS), further suggesting sexual dimorphism (Fig. 6B). Again, many inflammatory proteins showed greater differences between patients with PMOS and men, including IL-6, hsCRP, TNFα, plasminogen activator inhibitor-1, and several inflammatory glycoproteins, as well as permeability markers such as zinc finger protein, LBP, and GLP-2, among others (Supplementary Fig. S15). *Janibacter*, *Rheinheimera*, *Sphingomonas*, and *Acidovorax* showed sexual dimorphism being increased in both groups of women compared with men, whereas many metabolites—related in some cases to insulin resistance—were increased in men compared with both PMOS patients and control women (Supplementary Fig. S15). As an exception, proline was increased in women compared with men, particularly in those with PMOS (Supplementary Fig. S15).

## Discussion

### Integrative pathophysiological model

Our present multi-omics approach to the study of the complex interaction of PMOS and obesity provided new insights into this fascinating issue by integrating three different omics layers and identifying co-variation patterns between the serum metabolome and proteome with gut microbiota. Recently, multi-omics integration has been applied to the study of complex metabolic diseases such as obesity (Chakaroun *et al.*, 2026) and Type 2 diabetes (Li *et al.*, 2024). As with our present results, these studies demonstrated a more compressive understanding at the system level than the separate analysis of individual omics layers.

### Sexual dimorphism and androgenization pattern in women with PMOS

From a metabolomics perspective, our present results supported the existence of sexual dimorphism. The metabolome was the main contributor to the variability in latent Factor 1. This factor also correlated with androgen levels, indicating that the metabolome pattern differed between control women and men, a finding consistent with the existence of sexual dimorphism in metabolomic profiles (Escobar-Morreale *et al.*, 2023). But such sexual dimorphism was strongly modulated by obesity in an omics-specific manner.

Obesity attenuated sex-specific metabolic signatures while enhancing proteomic and gut microbiota sex differences. Men were characterized by less strict associations among metabolomic and proteomic variables, with a lesser impact of obesity on these features. Compared with control women, metabolite–metabolite associations in patients with PMOS weakened and were similar to those of men, a finding that might suggest a partial loss of metabolic coordination.

Consistent with this loss of metabolic coordination and with a partial masculinization of the metabolomic profile, branched-chain amino acids (BCAAs)—leucine, isoleucine, and valine—and their catabolic intermediates, 2-oxoisocaproic and 2-oxoisovaleric acids, were major contributors to MOFA+ Factors 1 and 2 and to PC1. Their accumulation suggested impaired BCAA catabolism, potentially related, at least in part, to androgen-mediated modulation of the activity of the branched-chain α-keto acid dehydrogenase complex (BCKDH) (Mora *et al.*, 2024). This interpretation agrees with earlier studies linking BCAA accumulation to insulin resistance and metabolic dysfunction in humans, including patients with PMOS (Newgard *et al.*, 2009; Lynch and Adams, 2014; Couto Alves *et al.*, 2017).

In this context, differences in body composition and energy homeostasis may have further contributed to these sex-specific patterns. Although men often exhibit lower insulin sensitivity and a less favorable metabolic profile, their higher skeletal muscle mass and enhanced capacity for ATP production may act as compensatory mechanisms that mitigate adverse metabolic outcomes (Karakelides *et al.*, 2010; Escobar-Morreale *et al.*, 2014; Li *et al.*, 2026). In contrast, androgen excess in women with PMOS does not appear to reproduce these functional adaptations, supporting the notion that androgenization does not translate into a protective, men-like metabolic phenotype (Escobar-Morreale *et al.*, 2014; Mauvais-Jarvis, 2024).

### Gut-inflammation axis in PMOS

Our results also highlighted the association of proinflammatory proteins and adipokines—the inflammasome—and of gut microbiota with both non-obese and obese patients with PMOS, characteristics apparently shared by obese control women and men (Insenser *et al.*, 2010; Escobar-Morreale *et al.*, 2011, 2017; Moncayo *et al.*, 2021).

Factor 3 was mainly driven by gut microbiota, and we observed inverse correlations with sex hormones and differences between control men and control women and with obese PMOS patients. These data again suggested a sexual dimorphic pattern in this omics layer that may be influenced by excess adiposity in a sex-dependent manner. As previously suggested (Santos-Marcos *et al.*, 2023), the interaction between sex hormones and high-fat environments may create distinct microbial signatures, which in our cohort may explain the specific dysbiosis observed in obese PMOS patients compared with their male counterparts. Of note, the almost perfect correlations observed among bacteria taxa would suggest loss of diversity in the composition of gut microbiota and the existence of a tight ecological and disturbed metabolic environment (gut dysbiosis) in obese individuals, and particularly in women with PMOS, in whom these microbiota disturbances may appear even in the absence of obesity (Insenser *et al.*, 2018).

Our findings may also support sexual dimorphism and that gut microbiota dysbiosis may translate in altered circulating proteomic patterns, consistent with its potential contribution to gut-derived inflammatory signaling in the context of gut permeability, particularly in metabolically compromised individuals such as those with PMOS and obesity (Martinez-Garcia *et al.*, 2024). Indeed, women with PMOS showed the strongest correlations between sCD14 (co-receptor of bacterial lipopolysaccharide) and gut genera independently of the bacteria clusters. While previous studies in PMOS have highlighted alterations in intestinal barrier markers like LBP (Banaszewska *et al.*, 2020), our multi-omics integration identified sCD14 as the primary link between the microbiota and systemic inflammation. This reinforces the hypothesis that metabolic endotoxemia, a mechanism well-characterized in experimental models (Cani *et al.*, 2007), might be a key driver of the proteomic signature in PMOS (Banaszewska *et al.*, 2020). Hence, in PMOS, the influence of gut microbiota on the modulation of the metabolic profile might be mediated through inflammatory proteins and adipokines, rather than through direct effects.

### Metabolic inflexibility and biomarker analysis

Correlation matrixes of proteomic variables indicated different patterns: those showing associations only with gut genera (sCD14, adiponectin, pentraxin-3, and IL-18); those that correlated with gut microbiota or metabolites depending on the subject subgroup (sLepR and FGF-23); and the remaining proteins that correlated only with metabolites.

The amino acid proline was the only metabolite consistently associated with inflammatory markers, gut permeability markers, and adipokines in all subgroups, particularly in obese women with and without PMOS and, to a lesser extent, in non-obese women with PMOS and in obese men. This pattern supported the concept that alterations in amino acid metabolism are closely linked to the metabolic dysregulation observed in PMOS (Phang *et al.*, 2008, 2010; Zhao *et al.*, 2012; Mayneris-Perxachs *et al.*, 2022; Huang *et al.*, 2026) where proline functions as a mitochondrial redox sensor and a key player in tissue remodeling and immune response under metabolic stress (Patriarca *et al.*, 2021). And because the associations of proline with inflammatory proteins were present in both non-obese and obese patients with PMOS, and was a major contributor to the separation between women with PMOS and men in PC2, our findings might suggest that proline levels in PMOS were not merely a metabolic byproduct, but a microenvironmental stress signal linking gut-derived inflammation with systemic metabolic dysfunction (Phang *et al.*, 2008, 2010).

Aside from proline, the most consistently altered metabolites were acetone, succinate, pyruvate, citrate, lactate, glycerol, acetate, isobutyric acid, betaine, serine, carnitine, choline, creatine, and tyrosine. These molecules are intimately linked to energy metabolism—carbohydrate and lipid oxidation—and may reflect impaired substrate switching, indicative of reduced metabolic flexibility. Indeed, metabolic inflexibility has been documented in women with PMOS, associated with insulin resistance, impaired lipid oxidation, and hyperandrogenism (Whigham *et al.*, 2014; Kim *et al.*, 2018; Rimmer *et al.*, 2020).

Even though several metabolites have been implicated in host–microbiota interactions and may show disease-specific alterations or correlations in PMOS (Hanna *et al.*, 2025), we observed no strong correlations between metabolites and gut bacteria in our cohort. However, our metabolomic panel was not specifically enriched for microbiota-derived metabolites, possibly limiting the detection of host–microbiota interactions (Liu *et al.*, 2022).

### The interplay between adiposity and androgen excess

The heatmaps indicated that women with PMOS as a whole presented with a pattern very similar to that of non-hyperandrogenic obese women. This finding suggested that PMOS exposed women to the same metabolic derangements as obesity does, without the need for weight excess. This agrees with the most recent conceptual framework on the ‘intimate relationship’ between adiposity and PMOS (Rosenfield and Dumesic, 2025), which posits a functional defect in adipose tissue function—rather than just excess fat mass—as a central driver of the syndrome’s metabolic and reproductive features, even in lean phenotypes.

Factor 2 was mainly driven by glycoproteins and pro-inflammatory proteins. Our observations were consistent with metabolic inflammation, abdominal adiposity dysfunction, and host–microbiota interactions as etiopathogenic mechanisms for the metabolic disturbances and low-grade chronic inflammation associated with PMOS. These findings are in conceptual agreement with the apparent paradox of sex-dependent effects of fat distribution on gonadal dysfunction (Borruel *et al.*, 2013; Escobar-Morreale *et al.*, 2014).

### From systems biology to personalized biomarkers in PMOS

The results derived from the MOFA+ model improved markedly those of PCA. In this context, our data indicated that multi-omics signatures may reflect co-variation patterns underlying biological pathways and the complex interplay linking sex hormones, adiposity, metabolism, inflammation, and gut microbiota. This integrative analysis revealed more subtle or variable-specific differences that might be crucial for understanding pathophysiological mechanisms and could serve as potential novel biomarkers or therapeutic targets.

Our study, however, was not free of limitations. The sample size was relatively small, which may reduce the statistical power to detect subtle associations. Technical and methodological constraints inherent to multi-omics analyses, including variability in measurement platforms and data preprocessing, could influence the results. Moreover, the metabolomics panel did not include the majority of gut-derived metabolites, and gut microbiota analyses did not reach metagenomics levels. Additionally, the women with PMOS included in this study had the classic hyperandrogenic phenotype of the syndrome, limiting the generalizability of the findings to other PMOS phenotypes. Lastly, causality cannot be inferred by the comparative nature of our present study.

Among the strengths, the multi-omics integration provided a comprehensive view of PMOS, combining metabolomic, proteomic, and gut microbial composition data to explore complex biological interactions that could have been missed using single-omics analyses. Our present findings might have clinical relevance, provided that the mechanisms underlying the observed associations are identified in future studies. The multi-omics patterns reported here may result in new hypotheses about the biological heterogeneity in PMOS and obesity-associated gonadal dysfunction and could help classify patients into multi-omics clusters. Such stratification could facilitate the design of personalized management strategies and targeted interventions in PMOS.

## Conclusion

Our multi-omics integration showed that PMOS is characterized by an androgen-driven metabolic reprogramming in which sexual dimorphism is reshaped in an obesity- and omics-specific manner. While metabolomic differences predominate in non-obese individuals, obesity shifts this pattern toward proteomic and gut microbiota alterations, particularly in women with PMOS. Notably, PMOS reproduces a metabolic profile similar to that of obesity even in the absence of excess weight, supporting the concept that adipose tissue dysfunction, rather than fat mass excess *per se*, is a central driver of the metabolic disturbances of the syndrome. Altogether, these findings highlight the complex interplay between androgen excess, adiposity, and host–microbiota interactions and provide a systems-level framework that may facilitate the identification of novel biomarkers and targeted therapeutic strategies in PMOS.

## Supplementary Material

hoag080_Supplementary_Data

## Data Availability

All data sets generated and/or analyzed during the current study are publicly available at https://github.com/eddgeag/integromics_2.
