# Supplementary material for: Multi-omics factor analysis v2 (MOFA+) reveals specific co-variation patterns in women with PMOS that are strongly influenced by obesity
Source: Hum Reprod Open. 2026 Sep 8;2026(4):hoag080. doi: 10.1093/hropen/hoag080 (PMC13614424; doi:10.1093/hropen/hoag080)

Supplementary Materials and Methods

Multi-Omics Factor Data Integration and Downstream Statistical Analysis

We applied the Multi-Omics Factor Analysis v2 (MOFA+) model to integrate and analyze multi-omics data (Argelaguet *et al.*, 2020). MOFA+ permitted the identification of underlying factors that drive variations across different omics layers (patterns of co-variation and co-occurrence), providing a comprehensive understanding of the biological systems involved.

We used a hybrid framework that combined an unsupervised exploratory and integrative modeling of the data with a confirmatory phase consisting of downstream and *post-hoc* analyses of phenotype-driven variation using Multi-Omics Factor Analysis v2 (MOFA+) (Argelaguet *et al.*, 2020). This method aimed to identify shared latent factors among three different omics layers. Metabolomics and proteomics data were preprocessed, scaled, and centered, while data from gut microbiota, due to its compositional nature, were normalized using the Dirichlet multinomial model in the ALDEx2 package (Fernandes *et al.*, 2014). No outliers were identified and there were no missing values in the dataset. Due to the normalization step, the model's likelihood was based on the Gaussian assumption.

The MOFA+ model integrated a total of 278 variables or features including: i) the serum metabolome consisting of 38 metabolites (Escobar-Morreale *et al.*, 2023b); ii) the proteomic profile including 27 circulating proteins and inflammatory glycoproteins (Fuertes-Martin *et al.*, 2019; Martinez-Garcia *et al.*, 2019; Martinez-Garcia *et al.*, 2020; Martinez-Garcia *et al.*, 2021; Martinez-Garcia *et al.*, 2024); and iii) the gut microbiota composed by 213 phylum and genera taxonomic groups (Insenser *et al.*, 2018). No prefiltered step was performed before integration. We run MOFA through 4 to 12 latent factors and 10 seeds. The best model was chosen as the one with the maximum ELBO, which was optimized. When the model was finished, diagnostic correlation plots between latent factors were computed to ensure that there was no correlation among them.

The MOFA+ model decomposes the observed data into latent factors (Z) and corresponding feature weights (W), as $X \approx ZW^T$, where Z represents latent factors capturing major

axes of variation across samples, and W contains feature loadings reflecting the contribution of each variable to the latent structure.

Then, a two-stage feature selection strategy was implemented that combined unsupervised latent-structure extraction with supervised statistical inference.

1) Selection based on latent factor loadings:

For each omics layer, features were initially selected based on their contribution to the latent factors. Specifically, features with absolute weights exceeding a predefined threshold ($|W| > \tau$) were retained. When multiple factors were considered, the maximum absolute loading across selected factors was used as $\max_f |W_{jf}| > \tau$, where f denotes the subset of factors of interest. This reconstruction reduces noise and isolates variation explained by the latent space. This step identified variables that contributed substantially to the underlying multi-omics structure inferred by MOFA+.

To focus on biologically relevant variation, omics data were reconstructed using the selected latent factors as $X^{(reconstructed)} = Z_f W_f^T$. The reconstruction of this signal allowed us to observe the variance explained by each factor and feature.

2) Selection based on Bayesian differential analysis by groups of subjects:

Each selected feature was subsequently evaluated for association with the main clinical independent variables - namely, obesity (non-obese and obese individuals) and subjects subgroups (control women, women with PMOS and control men) - and their interactions, using a Bayesian two-sample *t*-test framework. Empirical priors were derived from the data, including group-specific means and variances. For each contrast, the test yielded the Posterior mean difference, the Test statistic, and the *P* value. Multiple testing correction was performed using the Benjamini–Hochberg procedure.

Features were finally selected if they satisfied at least one of the above criteria: high contribution to latent factors ($|W| > 0.6$) or significant association in clinical contrasts (adjusted $p < \alpha$). This combined strategy allowed the identification of features that were either structurally relevant in the latent space or statistically associated with phenotypic variation. Following these

criteria, all metabolomic and proteomic features but only 8 taxa from gut microbiota were retained and included in subsequent analyses.

Following final selection, downstream analyses consisted of univariate (empirical Bayesian *t*-tests), two-way analysis of variance, bivariate analyses (correlation matrices with Pearson correlation coefficient), and multivariate Principal Component Analysis (PCA). These analyses were conducted over the latent factors and selected features of the reconstructed signal to determine how they relate to the main clinical independent variables. PCA allowed the visualization of the multivariate structure, and showed the contribution of each principal component to the maximum variance and the load of each feature within the PC by correlating the scores with the reconstructed signal.

All analyses used R v4.4.2 (R Foundation for Statistical Computing, Vienna, Austria; https://www.R-project.org/). $p < 0.05$ was considered statistically significant. The above code that contains the statistical strategy used as well as the description of packages and their corresponding versions is available at https://github.com/eddgeag/integromics_2.

References

Argelaguet, R., Arnol, D., Bredikhin, D., Deloro, Y., Velten, B., Marioni, J. C. and Stegle, O. (2020) MOFA+: a statistical framework for comprehensive integration of multi-modal single-cell data. Genome Biology, **21**, 111.

Escobar-Morreale, H. F., Martínez-García, M. Á., Insenser, M., Cañellas, N., Correig, X. and Luque-Ramírez, M. (2023) Serum metabolomics profiling by proton nuclear magnetic resonance reveals sexual dimorphism and masculinization of intermediate metabolism in women with polycystic ovary syndrome (PCOS). Biol Sex Diff, **14**, 21.

Fernandes, A. D., Reid, J. N. S., Macklaim, J. M., McMurrough, T. A., Edgell, D. R. and Gloor, G. B. (2014) Unifying the analysis of high-throughput sequencing datasets: characterizing RNA-seq, 16S rRNA gene sequencing and selective growth experiments by compositional data analysis. Microbiome, **2**, 15.

Fuertes-Martin, R., Moncayo, S., Insenser, M., Martinez-Garcia, M. A., Luque-Ramirez, M., Grau, N. A., Blanchar, X. C. and Escobar-Morreale, H. F. (2019) Glycoprotein A and B height-to-width ratios as obesity-independent novel biomarkers of low-grade chronic inflammation in women with polycystic ovary syndrome (PCOS). J Proteome Res, **18**, 4038-4045.

Insenser, M., Murri, M., Del Campo, R., Martinez-Garcia, M. A., Fernandez-Duran, E. and Escobar-Morreale, H. F. (2018) Gut microbiota and the polycystic ovary syndrome: Influence of sex, sex hormones, and obesity. J Clin Endocrinol Metab, **103**, 2552-2562.

Martinez-Garcia, M. A., Moncayo, S., Insenser, M., Alvarez-Blasco, F., Luque-Ramirez, M. and Escobar-Morreale, H. F. (2019) Metabolic cytokines at fasting and during macronutrient challenges: influence of obesity, female androgen excess and sex. Nutrients, **11**, E2566.

Martinez-Garcia, M. A., Moncayo, S., Insenser, M., Alvarez-Blasco, F., Luque-Ramirez, M. and Escobar-Morreale, H. F. (2021) Postprandial responses of circulating energy homeostasis mediators to single macronutrient challenges: influence of obesity and sex hormones. Food Funct, **12**, 1051-1062.

Martinez-Garcia, M. A., Quintero-Tobar, A., de Lope Quinones, S., Insenser, M., Fernandez-Duran, E., Escobar-Morreale, H. F. and Luque-Ramirez, M. (2024) Obesity and polycystic ovary syndrome influence on intestinal permeability at fasting, and modify the effect of diverse macronutrients on the gut barrier. Food Res Int, **186**, 114338.

Martinez-Garcia, M. A., Moncayo, S., Insenser, M., Montes-Nieto, R., Fernandez-Duran, E., Alvarez-Blasco, F., Luque-Ramirez, M. and Escobar-Morreale, H. F. (2020) Postprandial inflammatory responses after oral glucose, lipid and protein challenges: Influence of obesity, sex and polycystic ovary syndrome. Clin Nutr, **39**, 876-885.

Supplementary Figure S1: Weights of the variables contributing to the latent factors integrated by MOFA+

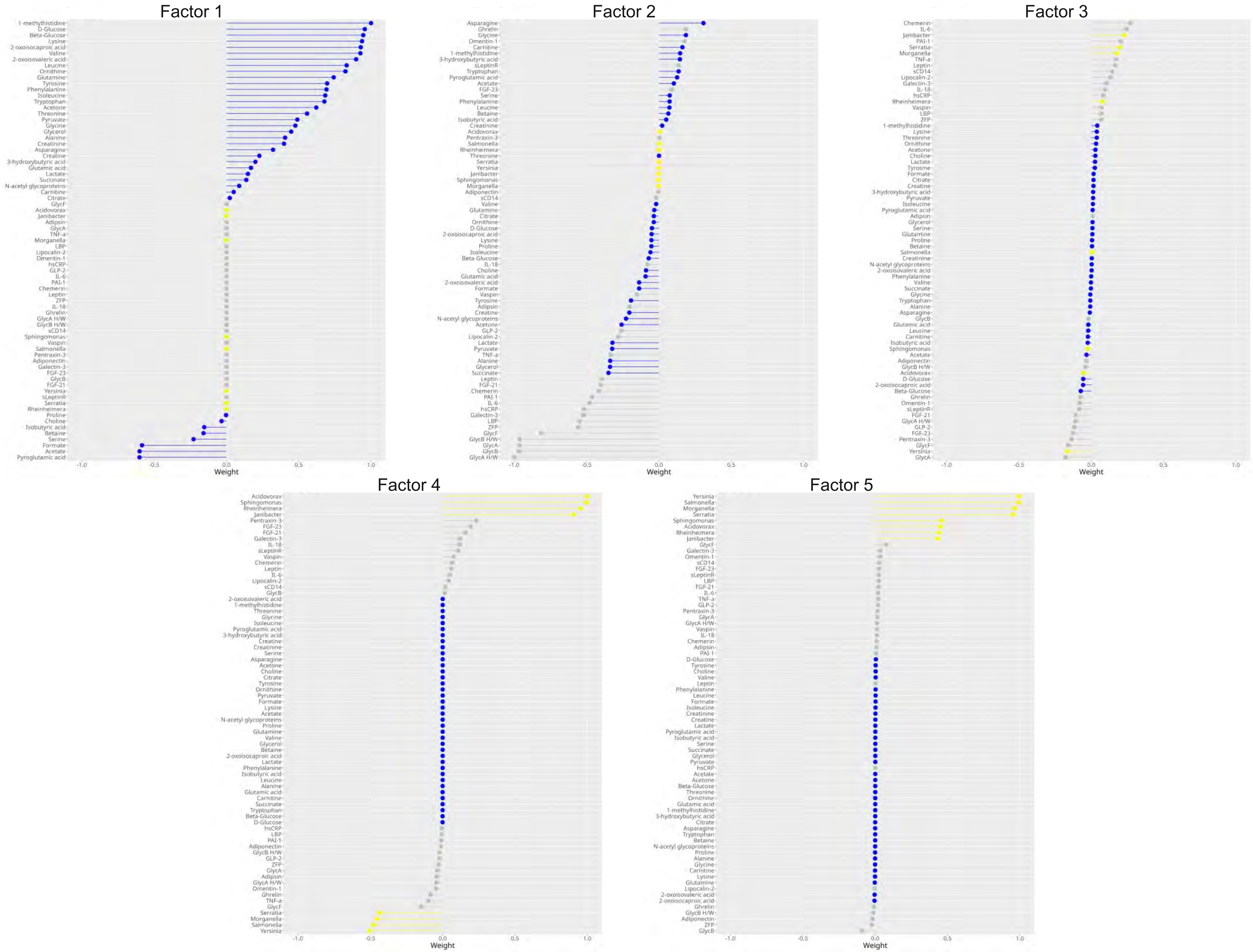

Supplementary Figure S2: Variables in the correlations heatmap of control women

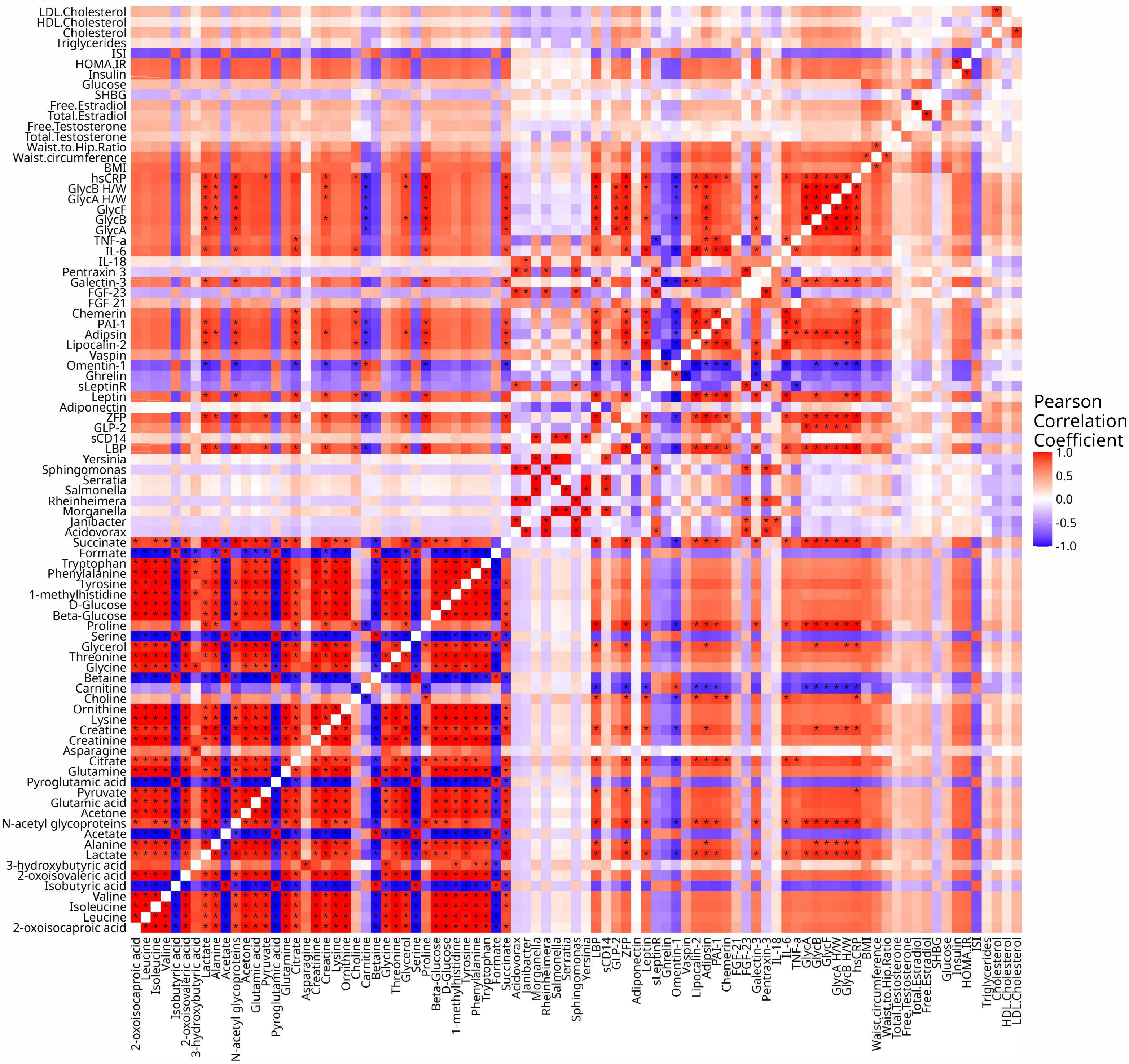

Correlation matrixes of the features derived from the reconstructed signal based on the MOFA+ integration model. Each cell represents the Pearson's correlation coefficient between two features.

Supplementary Figure S3: Variables in the correlations heatmap of PMOS patients

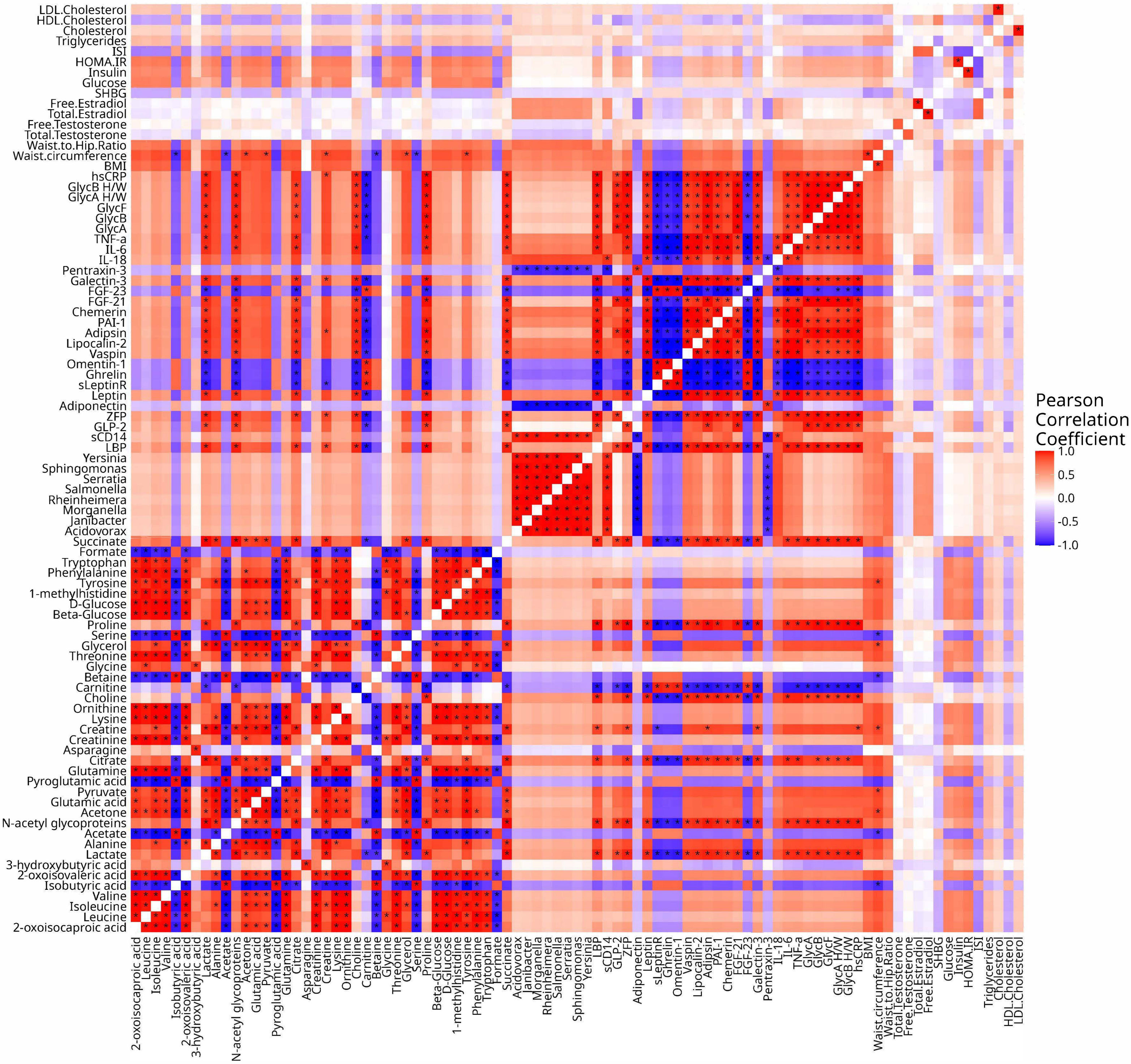

Correlation matrixes of the features derived from the reconstructed signal based on the MOFA+ integration model. Each cell represents the Pearson's correlation coefficient between two features.

Supplementary Figure S4: Variables in the correlations heatmap of control men

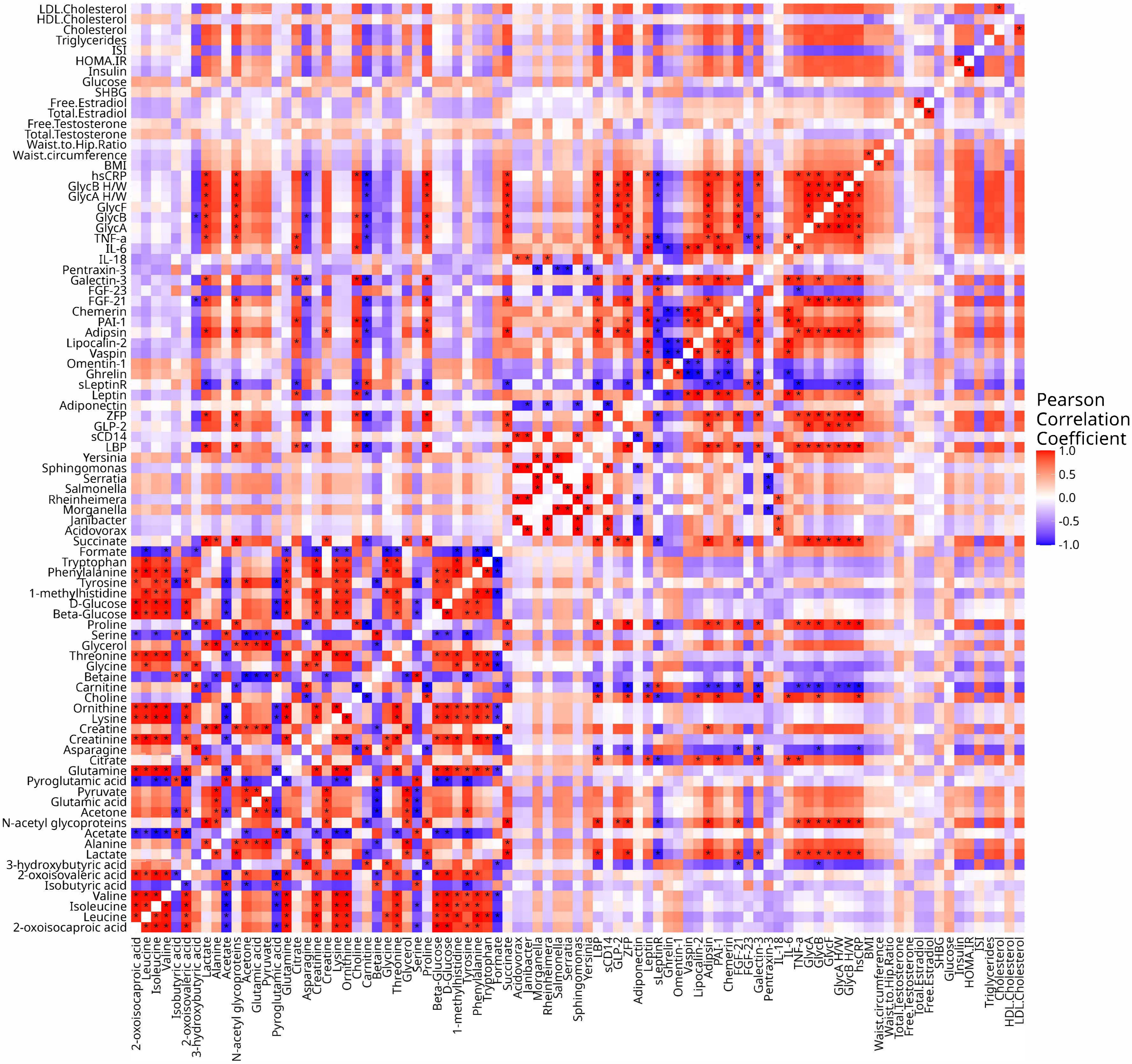

Correlation matrixes of the features derived from the reconstructed signal based on the MOFA+ integration model. Each cell represents the Pearson’s correlation coefficient between two features.

Supplementary Figure S5: Variables in the correlations heatmap of non-obese subjects

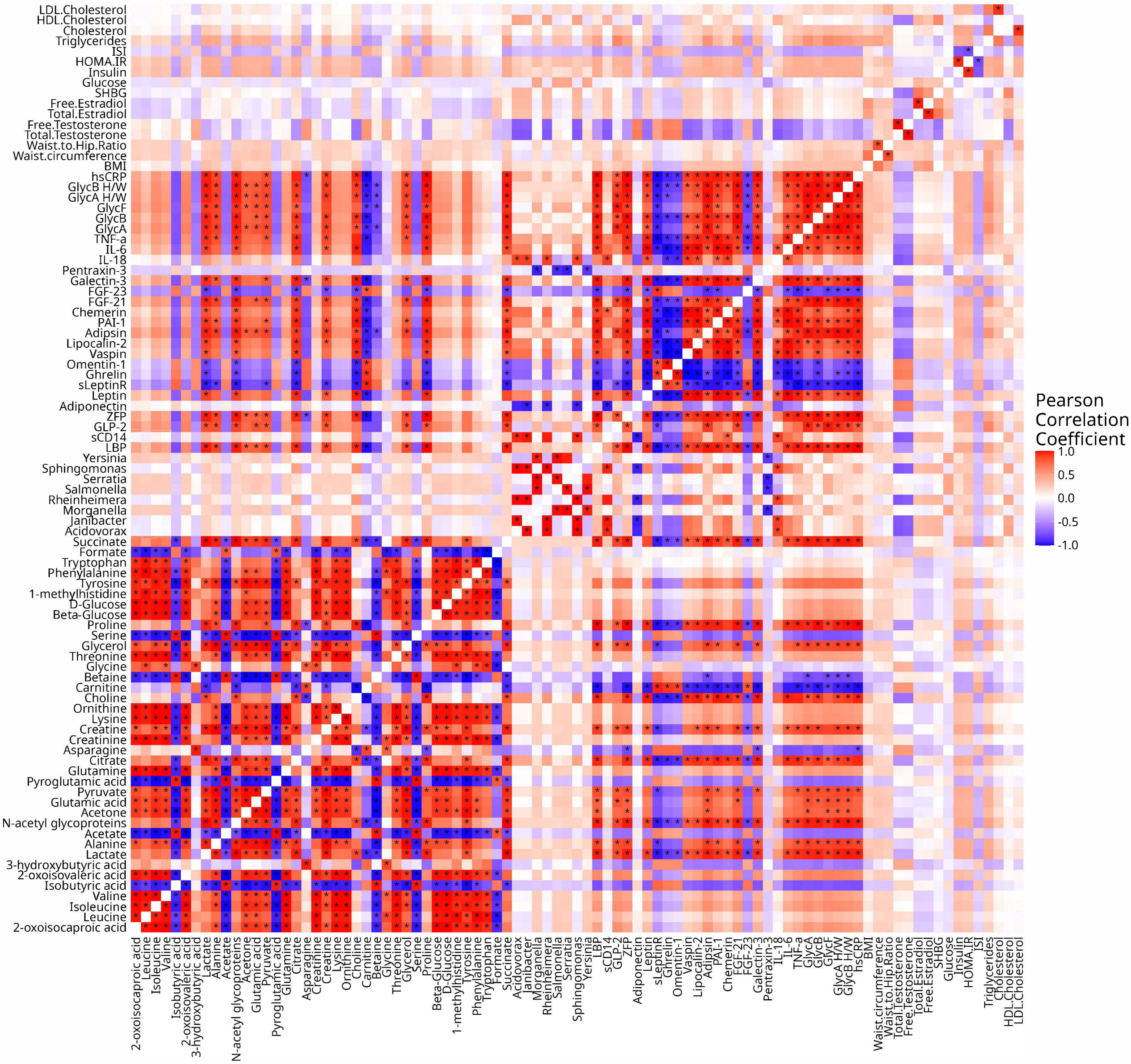

Correlation matrixes of the features derived from the reconstructed signal based on the MOFA+ integration model. Each cell represents the Pearson’s correlation coefficient between two features.

Supplementary Figure S6: Variables in the correlations heatmap of obese subjects

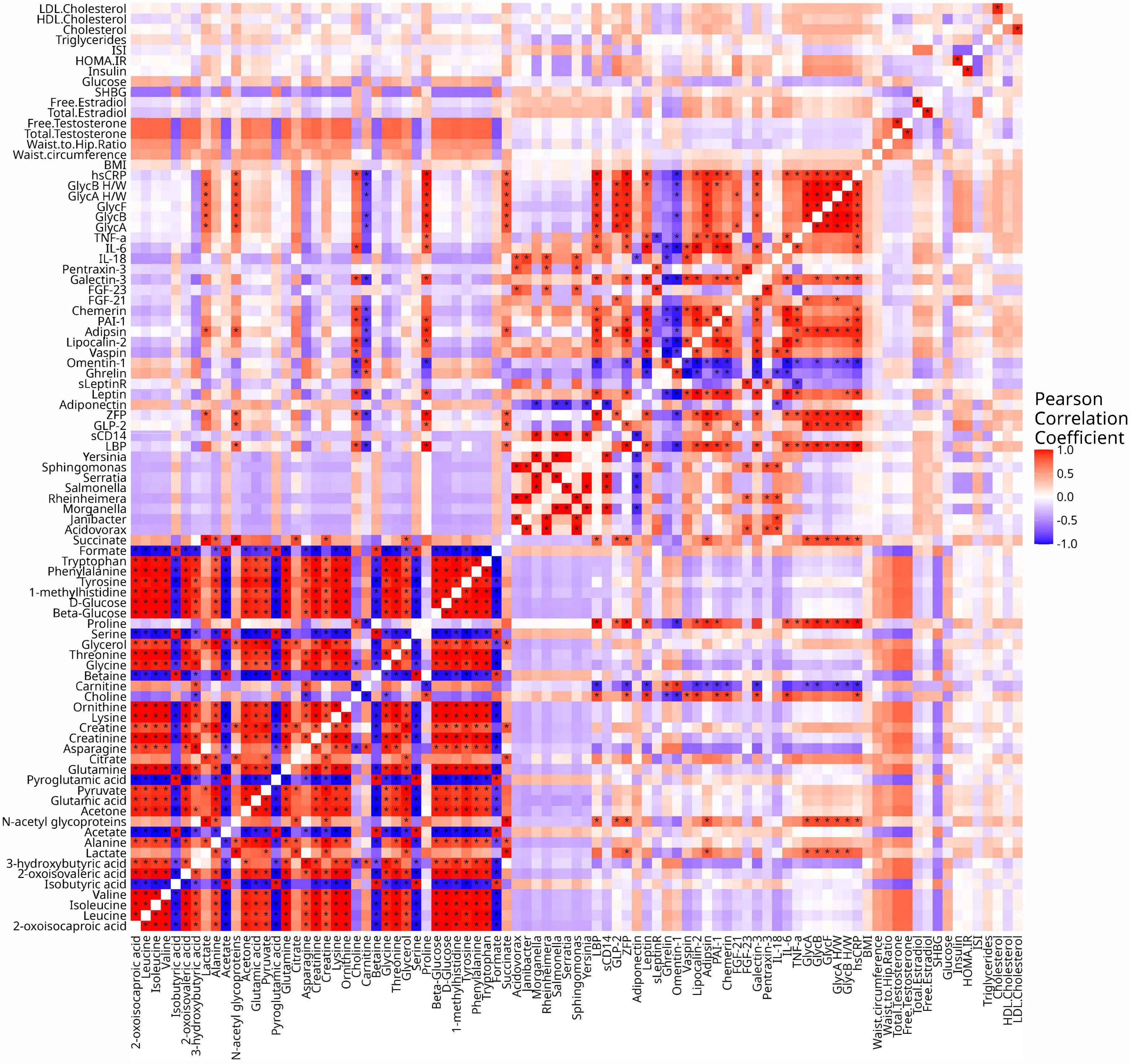

Correlation matrixes of the features derived from the reconstructed signal based on the MOFA+ integration model. Each cell represents the Pearson’s correlation coefficient between two features.

Supplementary Figure S7: Variables in the correlations heatmap of non-obese control women

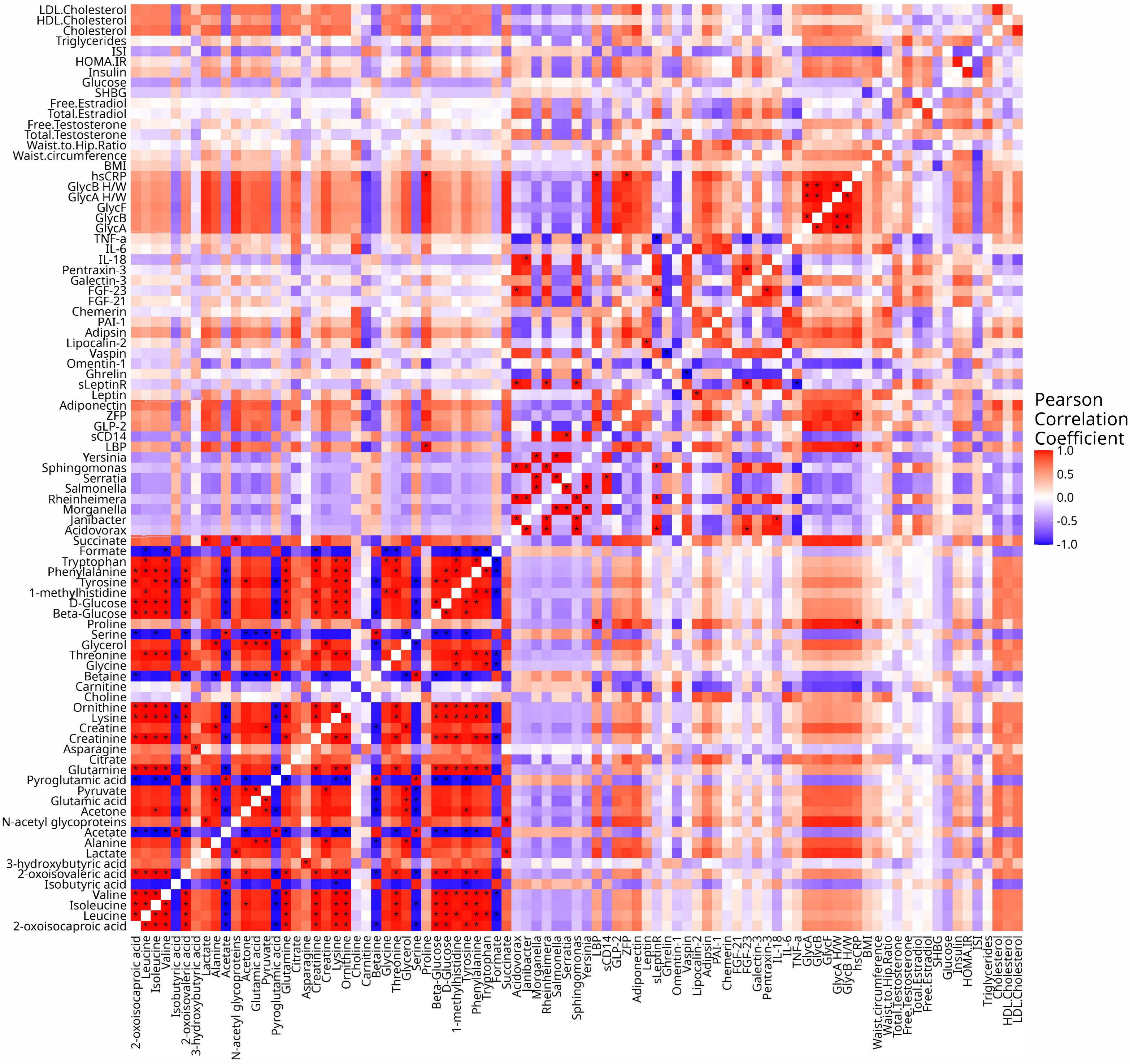

Correlation matrixes of the features derived from the reconstructed signal based on the MOFA+ integration model. Each cell represents the Pearson's correlation coefficient between two features.

Supplementary Figure S8: Variables in the correlations heatmap of non-obese PMOS patients

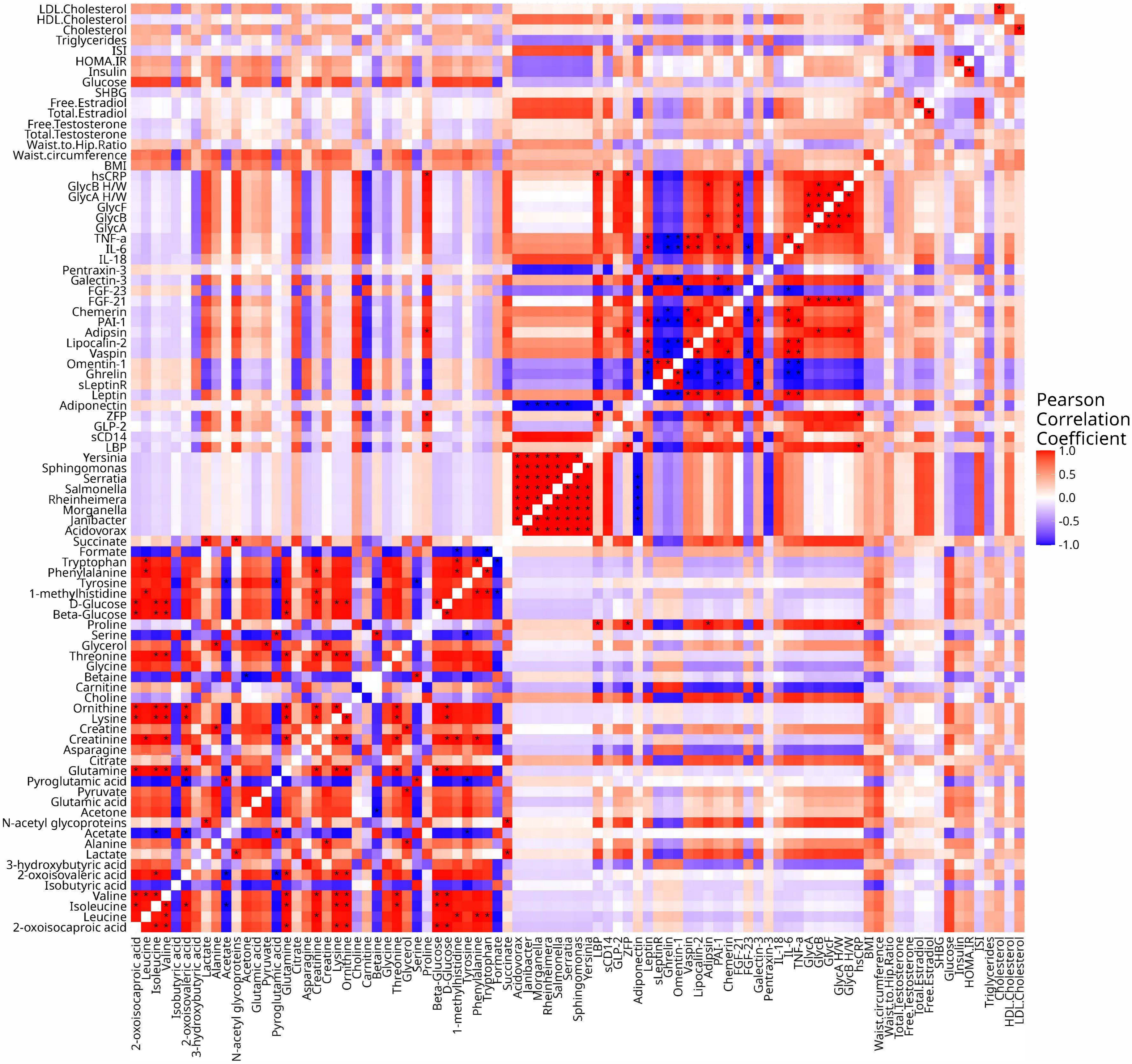

Correlation matrixes of the features derived from the reconstructed signal based on the MOFA+ integration model. Each cell represents the Pearson's correlation coefficient between two features.

Supplementary Figure S9: Variables in the correlations heatmap of non-obese control men

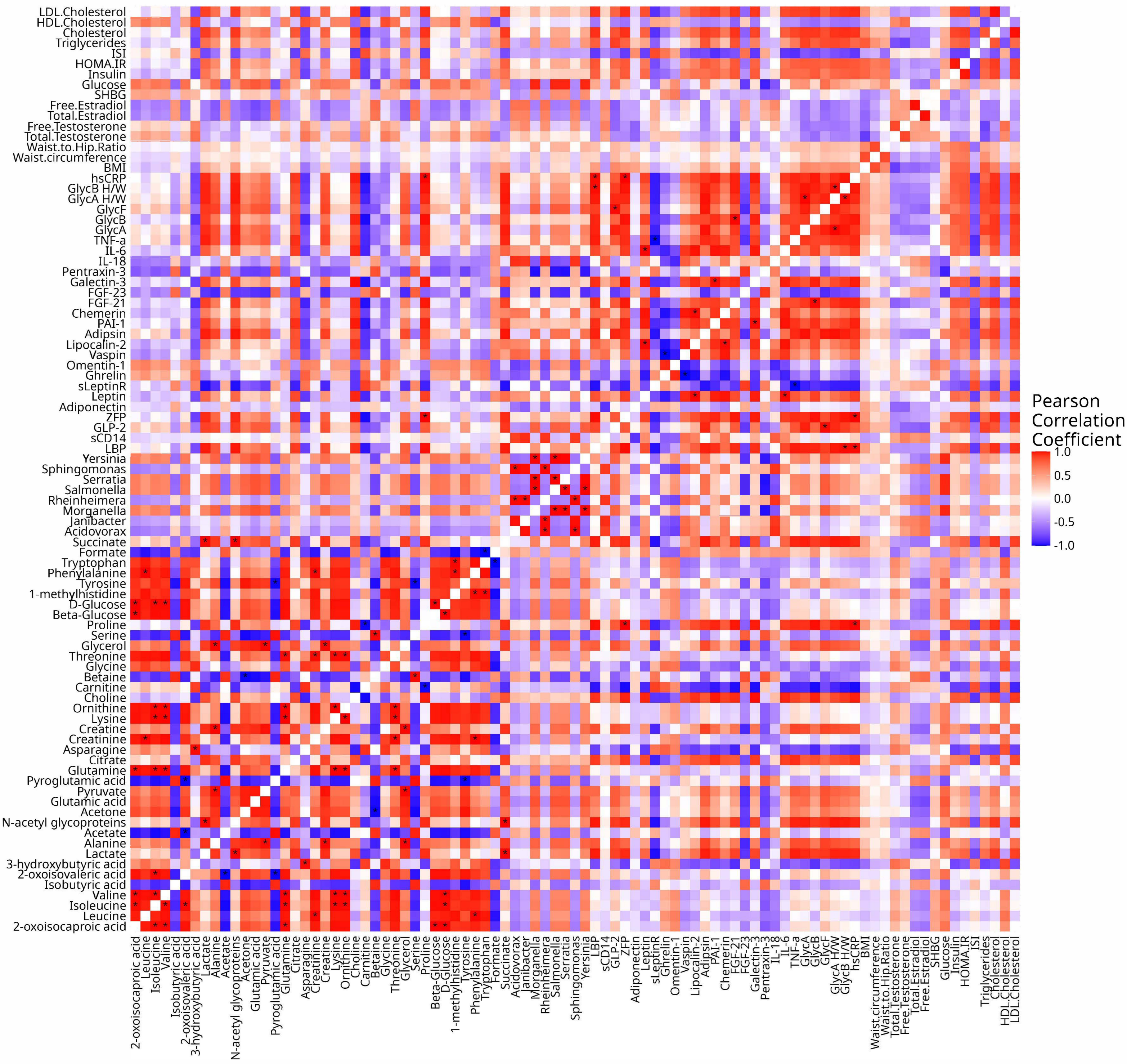

Correlation matrixes of the features derived from the reconstructed signal based on the MOFA+ integration model. Each cell represents the Pearson's correlation coefficient between two features.

Supplementary Figure S10: Variables in the correlations heatmap of obese control women

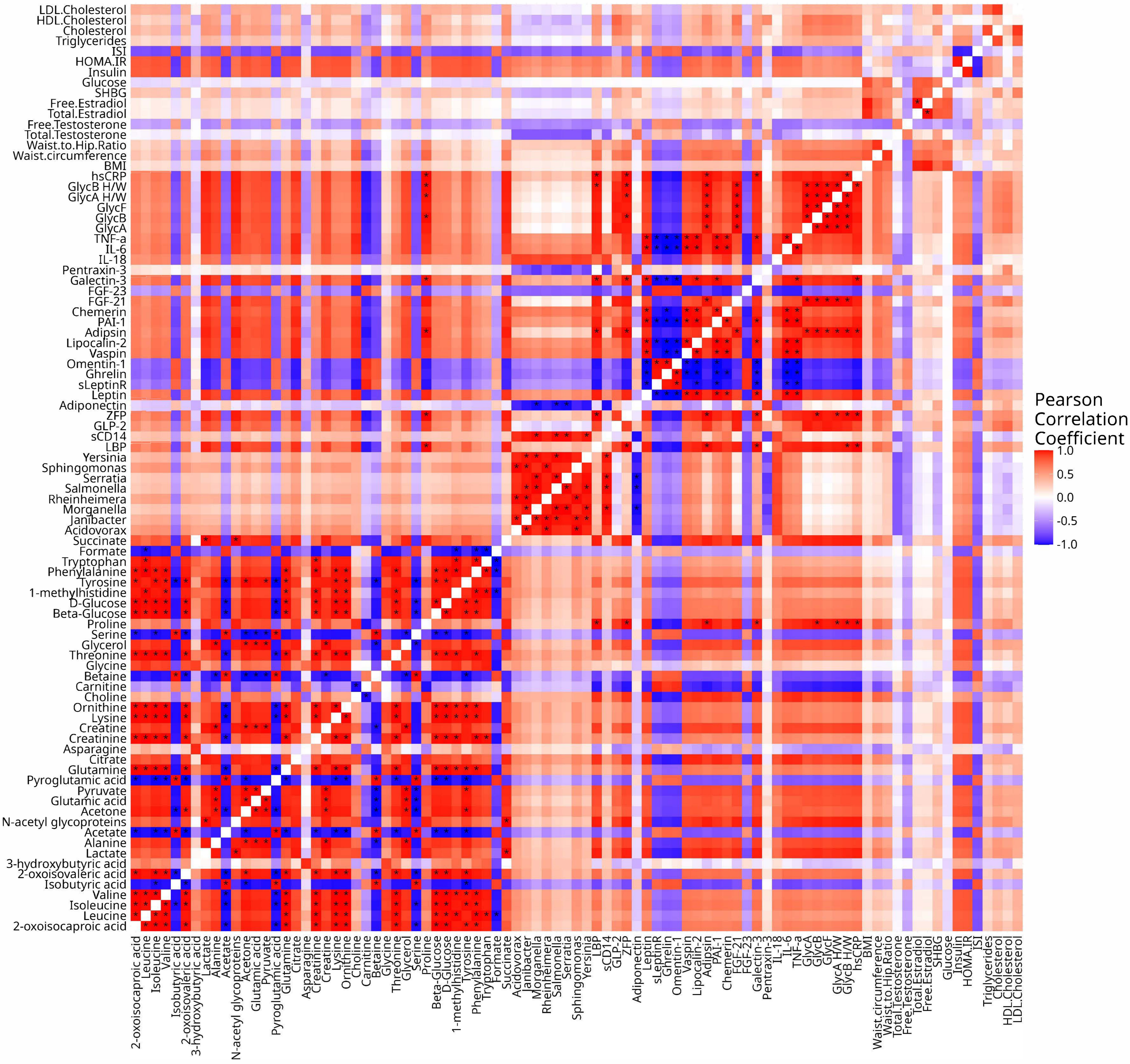

Correlation matrixes of the features derived from the reconstructed signal based on the MOFA+ integration model. Each cell represents the Pearson's correlation coefficient between two features.

Supplementary Figure S11: Variables in the correlations heatmap of obese PMOS patients

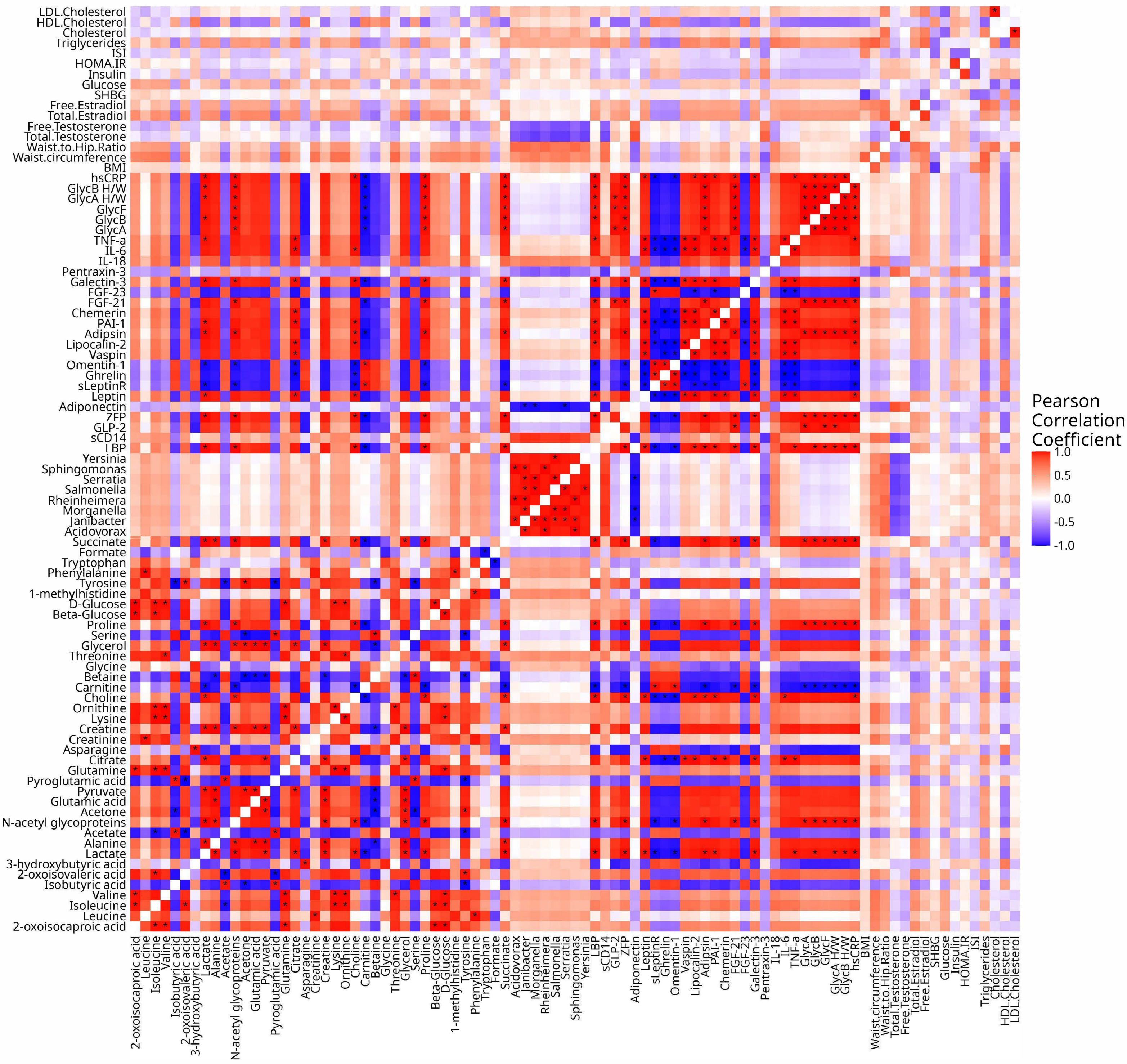

Correlation matrixes of the features derived from the reconstructed signal based on the MOFA+ integration model. Each cell represents the Pearson's correlation coefficient between two features.

Supplementary Figure S12: Variables in the correlations heatmap of obese control men

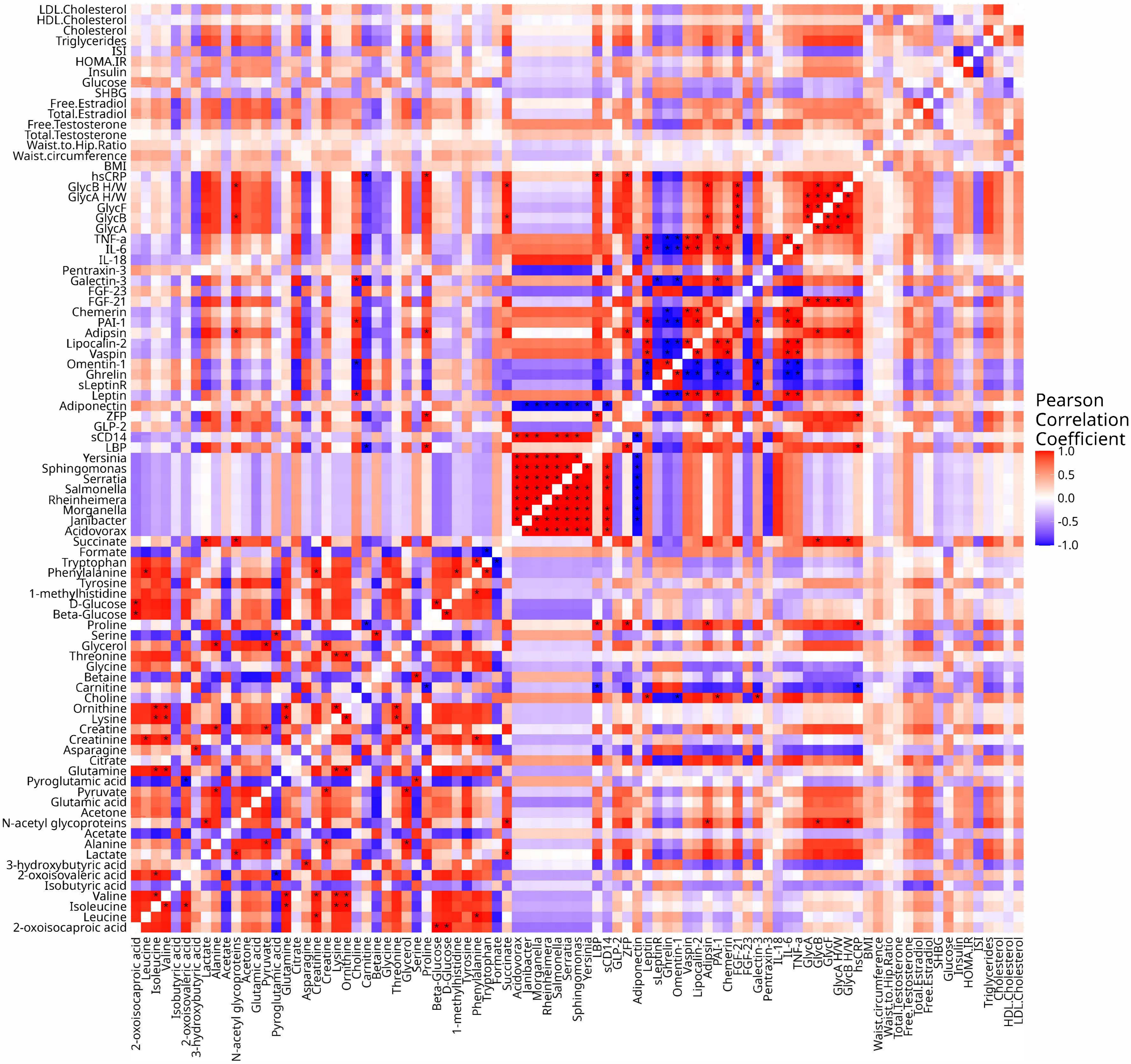

Correlation matrixes of the features derived from the reconstructed signal based on the MOFA+ integration model. Each cell represents the Pearson's correlation coefficient between two features.

Supplementary Figure S13: Variables contributing to PC1 and PC2 with weights

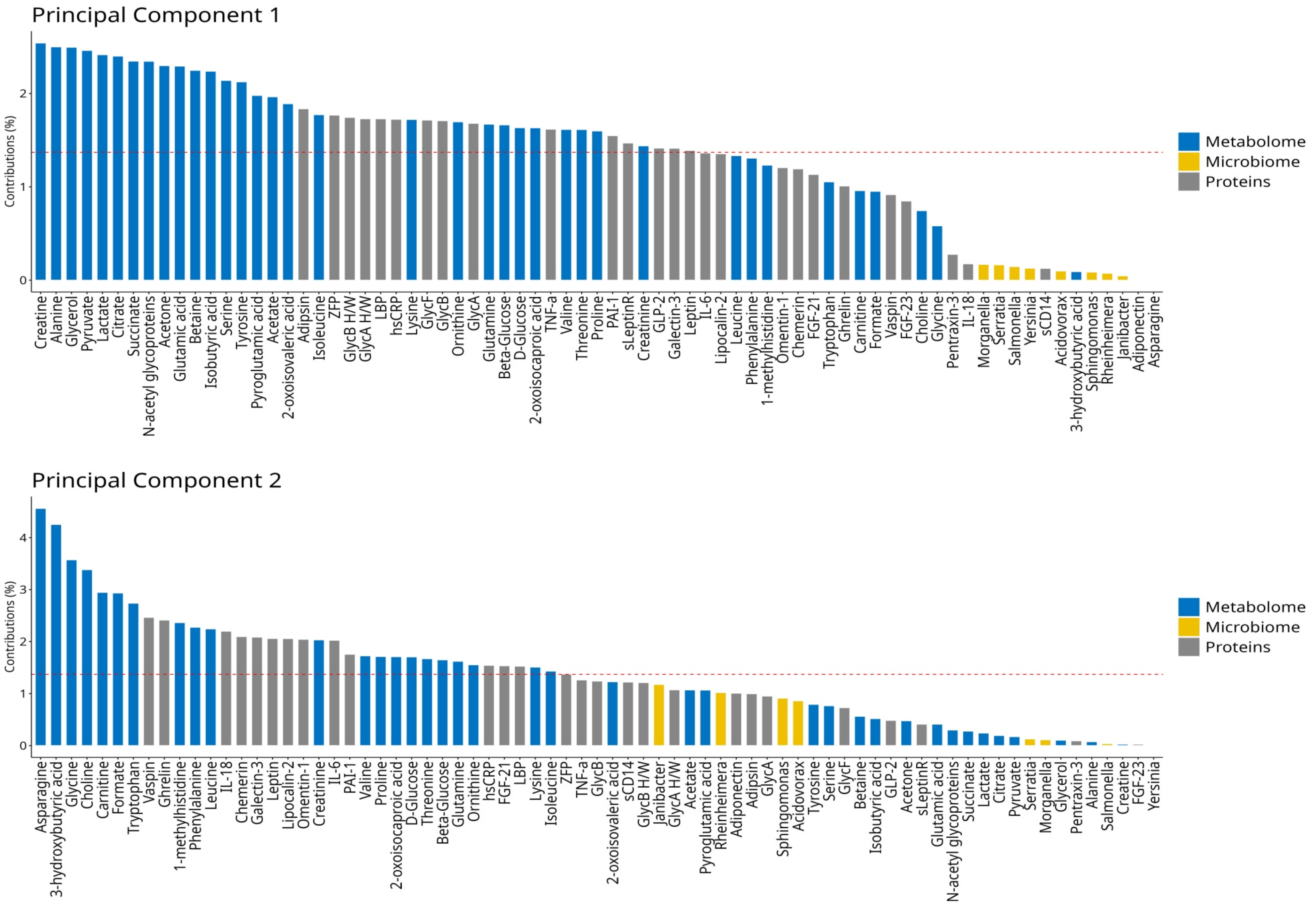

Supplementary Figure S14: Variables contributing to the differences among subgroups in PC1

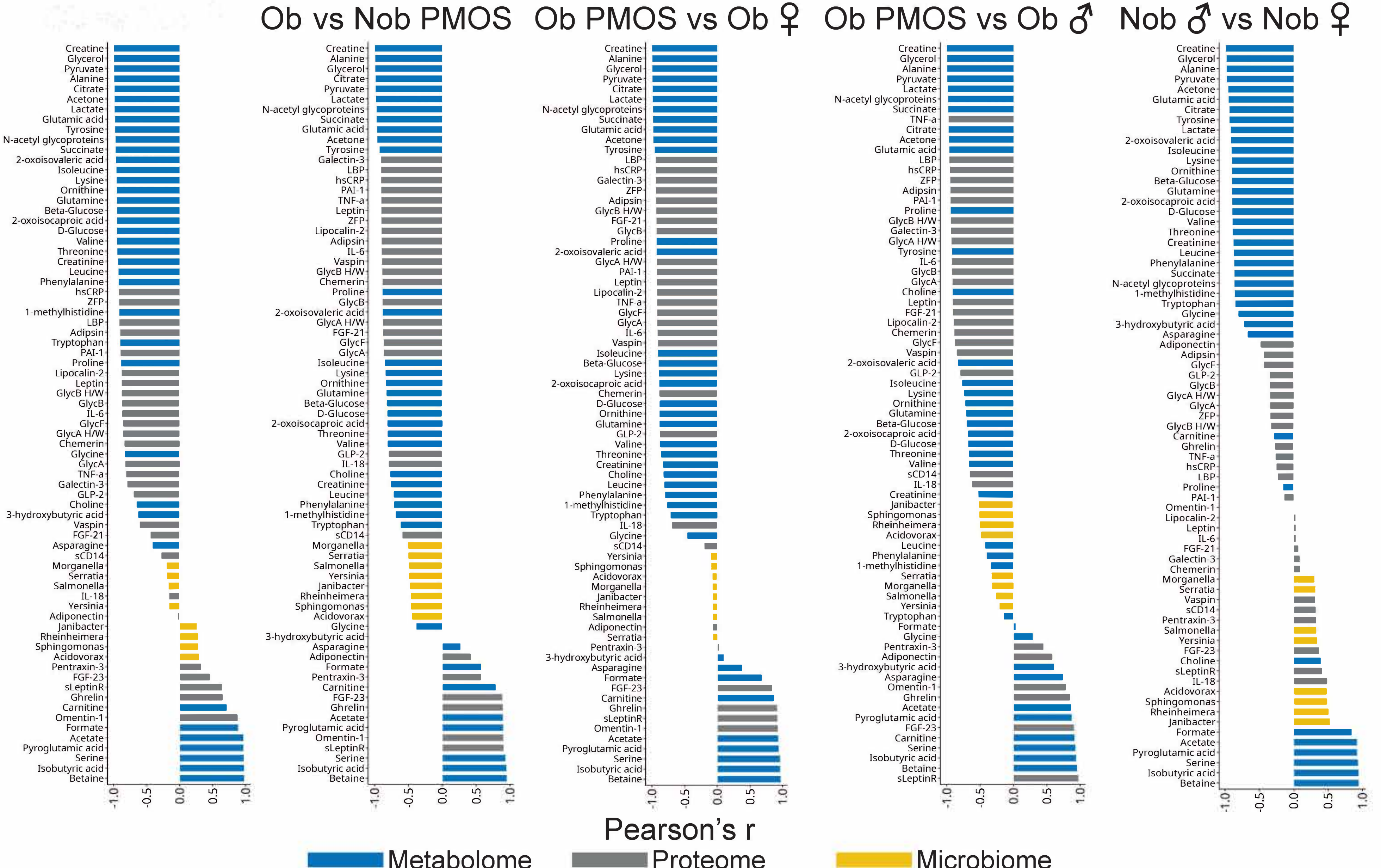

Supplementary Figure S15: Variables contributing to the differences among subgroups in PC2

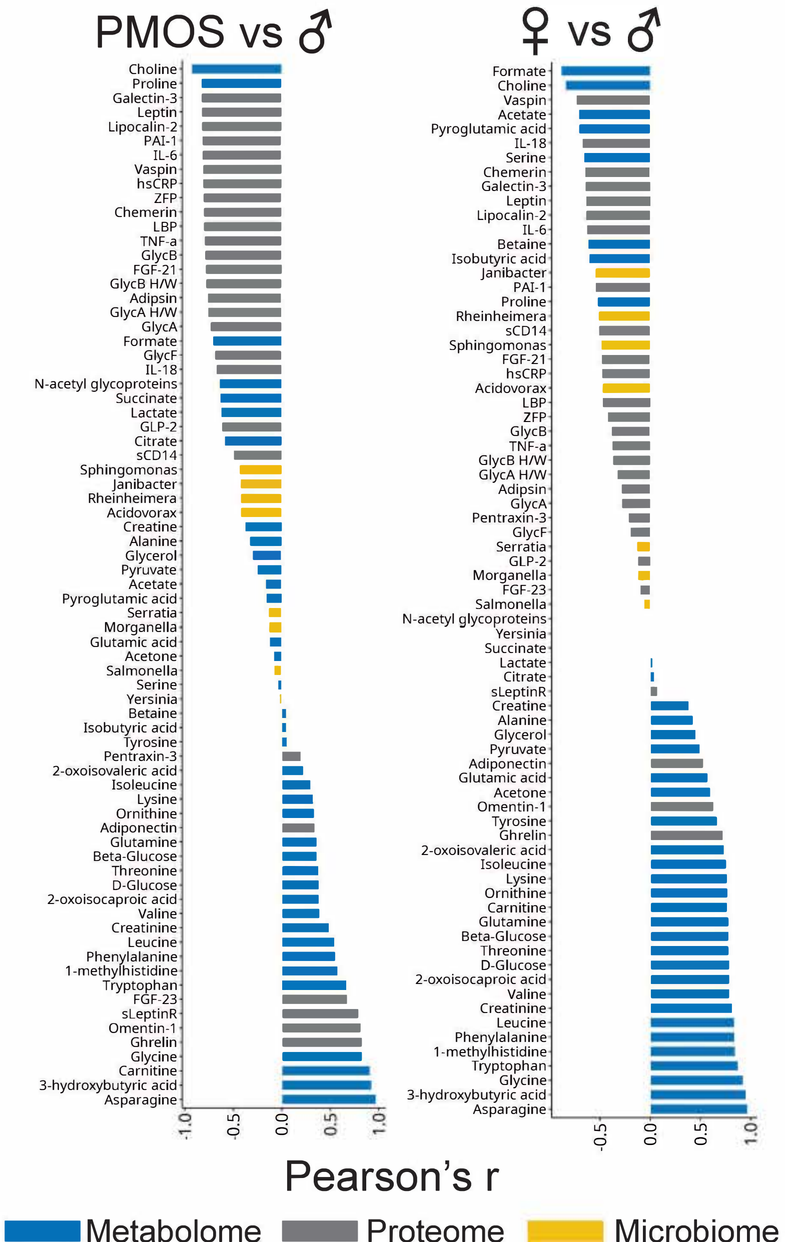

Supplement: Supplementary file 1 [file hoag080_supplementary_data.zip › Supplementary Data Text & Figures HROPEN-26-0234.R2.pdf]
